# Consistency across multi‐omics layers in a drug‐perturbed gut microbial community

**DOI:** 10.15252/msb.202311525

**Published:** 2023-07-24

**Authors:** Sander Wuyts, Renato Alves, Maria Zimmermann‐Kogadeeva, Suguru Nishijima, Sonja Blasche, Marja Driessen, Philipp E Geyer, Rajna Hercog, Ece Kartal, Lisa Maier, Johannes B Müller, Sarela Garcia Santamarina, Thomas Sebastian B Schmidt, Daniel C Sevin, Anja Telzerow, Peter V Treit, Tobias Wenzel, Athanasios Typas, Kiran R Patil, Matthias Mann, Michael Kuhn, Peer Bork

**Affiliations:** ^1^ European Molecular Biology Laboratory Heidelberg Germany; ^2^ Medical Research Council Toxicology Unit Cambridge UK; ^3^ Department of Proteomics and Signal Transduction Max Planck Institute of Biochemistry Martinsried Germany; ^4^ Cellzome GlaxoSmithKline R&D Heidelberg Germany; ^5^ Proteomics Program, NNF Center for Protein Research, Faculty of Health Sciences University of Copenhagen Copenhagen Denmark; ^6^ Max Delbrück Centre for Molecular Medicine Berlin Germany; ^7^ Yonsei Frontier Lab (YFL) Yonsei University Seoul South Korea; ^8^ Department of Bioinformatics, Biocenter University of Würzburg Würzburg Germany; ^9^ Present address: MOSTMICRO Unit, Instituto de Tecnologia Quimica e Biologica Universidade Nova de Lisboa Oeiras Portugal; ^10^ Present address: Institute for Biological and Medical Engineering, Schools of Engineering, Medicine and Biological Sciences Pontificia Universidad Catolica de Chile Santiago Chile

**Keywords:** metabolomics, metagenomics, metaproteomics, metatranscriptomics, microbiology, Microbiology, Virology & Host Pathogen Interaction, Proteomics

## Abstract

Multi‐omics analyses are used in microbiome studies to understand molecular changes in microbial communities exposed to different conditions. However, it is not always clear how much each omics data type contributes to our understanding and whether they are concordant with each other. Here, we map the molecular response of a synthetic community of 32 human gut bacteria to three non‐antibiotic drugs by using five omics layers (16S rRNA gene profiling, metagenomics, metatranscriptomics, metaproteomics and metabolomics). We find that all the omics methods with species resolution are highly consistent in estimating relative species abundances. Furthermore, different omics methods complement each other for capturing functional changes. For example, while nearly all the omics data types captured that the antipsychotic drug chlorpromazine selectively inhibits Bacteroidota representatives in the community, the metatranscriptome and metaproteome suggested that the drug induces stress responses related to protein quality control. Metabolomics revealed a decrease in oligosaccharide uptake, likely caused by Bacteroidota depletion. Our study highlights how multi‐omics datasets can be utilized to reveal complex molecular responses to external perturbations in microbial communities.

## Graphical abstract

Multi‐omics analysis allows for an in‐depth view of the molecular dynamics of a microbial ecosystem. The study evaluates if different omics data types show similar results or if each of them provides unique insights into the dynamics of a drug‐perturbed microbial ecosystem.

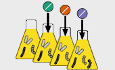

## Introduction

The human gut microbiota is a complex community of microorganisms, which is affected by endogenous and environmental factors such as host genotype, diet, drug treatment and disease status, and in turn, influences host health and disease progression (Kau *et al*, 2011; Cho & Blaser, 2012; Cani, 2018; Durack & Lynch, 2018; Schmidt *et al*, 2018; Lindell *et al*, 2022). Currently, insights into the structure and function of the microbiota community mainly come from 16S rRNA gene profiling and shotgun metagenomics. While 16S rRNA amplicon sequencing offers a cost‐efficient way to assess bacterial abundance at a higher taxonomic level, whole‐genome shotgun metagenomics resolves the abundance of species and strains, together with the functional potential they encode (Quince *et al*, 2017; Almeida *et al*, 2019; Pasolli *et al*, 2019). In addition, gene and protein expression and metabolite abundance in the community can be quantified with metatranscriptomics (Bashiardes *et al*, 2016), metaproteomics (Zhang & Figeys, 2019) and metabolomics (Zierer *et al*, 2018; Han *et al*, 2021), respectively. Ultimately, the combination of these methods should enable the integration of the major molecular layers of the cell, resulting in a more complete picture of the microbiome (Jansson & Baker, 2016; Heintz‐Buschart & Wilmes, 2018). Several studies have shown how a combination of two or more of these omics methods could lead to novel insights regarding the dynamics and inner workings of a microbial community (Heintz‐Buschart *et al*, 2016; Lloyd‐Price *et al*, 2017; Salazar *et al*, 2019; Taylor *et al*, 2020). While multi‐omics measurements provide information across molecular layers, their comprehensive integration remains challenging. One challenge is the limited knowledge about the concordance of different measurements in complex *in natura* settings in the absence of ground truth. Another challenge in comparing and integrating multi‐omics datasets is the difference in their dynamics in response to perturbations. Although metabolite changes occur on a time scale of seconds, transcriptional changes usually occur on a time scale of minutes, while protein abundance changes take the longest to respond to a perturbation (Gerosa & Sauer, 2011; Choi *et al*, 2020).

Synthetic microbial communities have been increasingly used to obtain a better understanding of the dynamics and species–species interactions (Goldford *et al*, 2018; preprint: Cheng *et al*, 2021). Compared with a natural gut microbiota, these synthetic communities have lower complexity, higher controllability and reproducibility and a well‐defined composition at the strain level, at the cost of being simplified representations of natural ecosystems (Roy *et al*, 2014; Aranda‐Díaz *et al*, 2022; Weiss *et al*, 2022). Yet, they do offer advantages over single species studies, as single species' behaviour can significantly differ in mono‐culture compared with co‐culture (D'hoe *et al*, 2018).

The complex interactions between the gut microbiota and non‐antibiotic drugs have been elucidated from large‐scale human studies and high‐throughput laboratory experiments (Rizkallah *et al*, 2010; Forslund *et al*, 2015, 2021; Spanogiannopoulos *et al*, 2016; Wilson & Nicholson, 2017; Zimmermann *et al*, 2021). This relationship is bidirectional, as drugs can influence microbiome composition (Jackson *et al*, 2018; Maier *et al*, 2018; Vich Vila *et al*, 2020; Vieira‐Silva *et al*, 2020), while the gut microbiota can have an impact on a drug's efficacy and toxicity by altering its chemical structure (Zimmermann *et al*, 2019a,b; Javdan *et al*, 2020; Klünemann *et al*, 2021). The emerging knowledge on drug–microbiota interactions has the potential to influence the future of drug development and personalised medicine (Doestzada *et al*, 2018; Weersma *et al*, 2020; Maier *et al*, 2021; Zimmermann *et al*, 2021).

We therefore set out to answer the following three questions: How do the different omics methods perform in capturing dynamic changes in microbial communities in response to perturbations? Can we identify the drug's mechanism of action on the bacteria, or the bacteria's defensive responses? In which time frame do drugs cause perturbations on the bacteria, as visible in genomes, transcripts, proteins and metabolites? To this end, we designed a controlled time‐course experiment with a synthetic community of 32 human gut representatives (Tramontano *et al*, 2018) in response to three drugs from diverse indication areas: chlorpromazine (antipsychotic), metformin (antidiabetic) and niclosamide (anthelmintic), which were previously reported to impair growth of several gut bacteria (Maier *et al*, 2018). We followed the response of the defined community to the three non‐antibiotic drugs over 4 days on the structural and functional levels across multi‐omics layers, based on 16S rRNA gene, metagenome, metatranscriptome, metaproteome and untargeted metabolome profiling.

## Results

### Establishment of a synthetic community for drug perturbations

**Figure 1:**
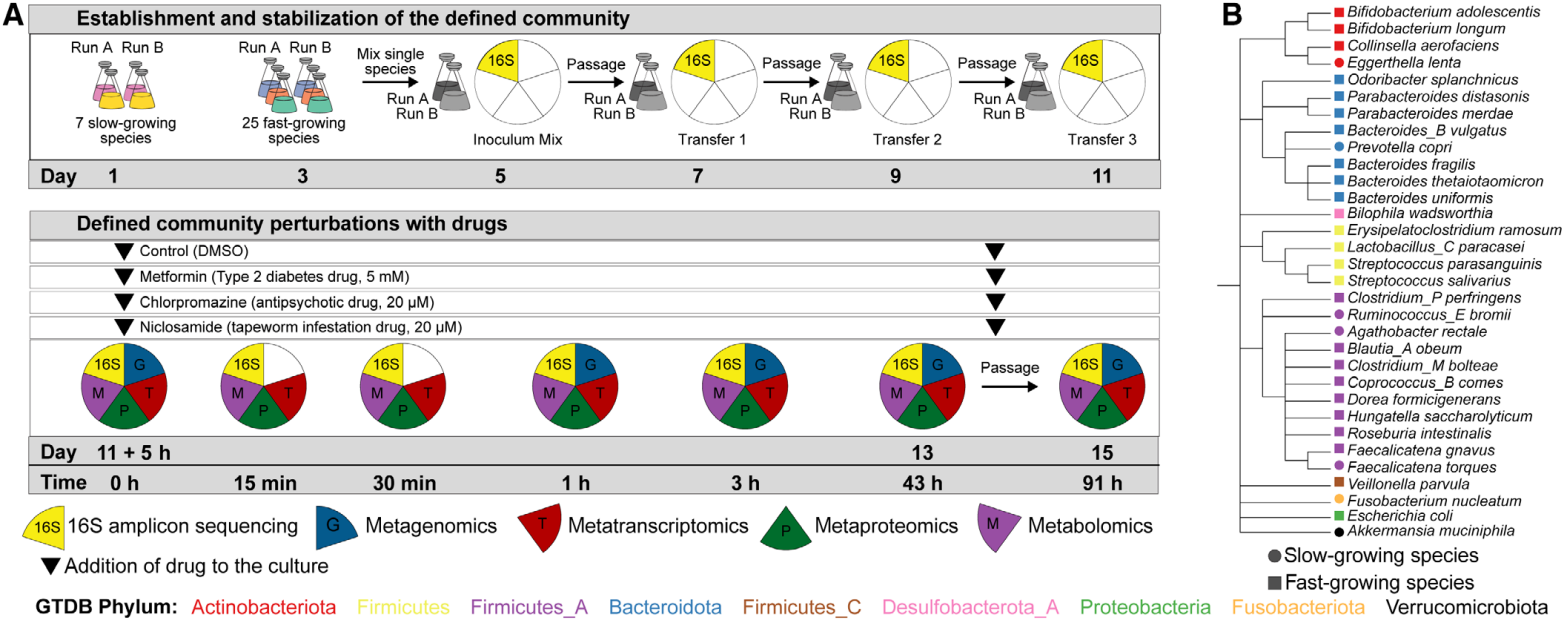
Experimental design and species used in this study Schematic overview of the experimental design.Species cladogram constructed by pruning the relevant species from the GTDB species cladogram (release 95).

**Figure EV1:**
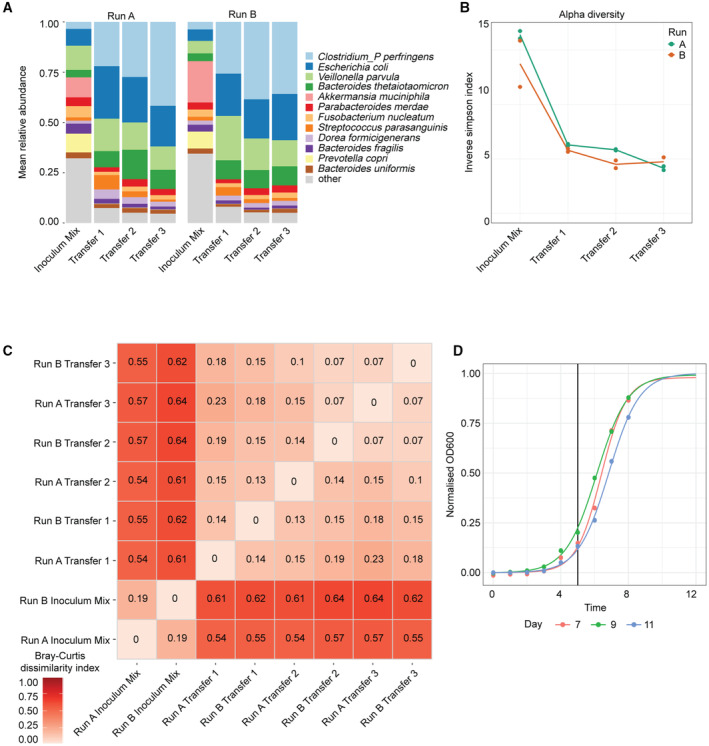
Establishment of a stable microbial community after three community transfers Relative abundance of the community members during the community transfer phase prior to drug treatment.Alpha‐diversity measurements during the community transfer phase prior to drug treatment.The Bray–Curtis dissimilarity values for pairwise comparison of community compositions during the community transfer phase prior to drug treatment.Growth curves were measured every hour during community establishment. We fit a sigmoid function to the measurements per day, and normalised the resulting OD curves. Based on the observed growth curve, we chose to treat the community after 5 h (black vertical line) so that the tightly spaced time points within 3 h are all within the exponential phase.

To investigate microbial community response to drug perturbations in a controlled system across five omics layers, we combined 32 human gut microbiome representatives (Tramontano *et al*, 2018) and exposed this community to three different non‐antibiotic drugs (Fig 1A; Appendix Table S1). The complete experiment was performed twice (run A and run B) as biological replicates, starting from the initial community assembly step from single bacterial cultures. More specifically, seven slow‐growing species (inoculated on day 1) were combined with 25 fast‐growing species (inoculated on day 3) on day 5 to form a synthetic community (Fig 1A and B). In order to ensure stable community composition, we performed three culture passages by growing the mixed culture for 48 h and transferring 1% of total volume to a fresh culture medium. Samples for 16S rRNA amplicon sequencing were taken immediately after combining the strains (Inoculum mix) and after each passage (Transfers 1–3) to evaluate the stabilisation of the community (Fig 1A; top row). We found that in both runs of the experiment the community reached a stable composition with four highly abundant species after three transfers (relative abundance > 10% for *Escherichia coli*, *Clostridium perfringens*, *Veillonella parvula* and *Bacteroides thetaiotaomicron*, Fig EV1A). The Bray–Curtis dissimilarity index showed that both runs were highly similar after the third transfer (Fig EV1C).

After stabilisation, in each run the community perturbation was performed in duplicate during exponential growth (i.e., 5 h after passaging, as determined by optical density [OD] measurements on the previous transfer; Fig EV1D) by addition of one of the following drugs: (i) 5 mM metformin, a type 2 diabetes drug, (ii) 20 μM chlorpromazine, an antipsychotic drug, or (iii) 20 μM niclosamide, an anthelmintic drug (Fig 1A), while DMSO was used as a control. These are in the range of the estimated colon concentrations, which are available for metformin (1.5 mM) and chlorpromazine (25 μM) (Maier *et al*, 2018). We chose a higher concentration for metformin based on the reported intestinal concentration and previous data on metformin concentrations sufficient to impair growth of gut microbiota members *in vitro* (Bailey *et al*, 2008b; Maier *et al*, 2018). The communities were sampled right before the addition of the drugs and 15 min, 30 min, 1 h and 3 h following the drug perturbation (Fig 1A). These time points were chosen to elucidate the early response of the bacterial community to the drug treatment. After 43 h, an additional sample was taken, and the communities were transferred into a fresh culture medium containing the drugs at initial concentrations. A final sample was taken 48 h after this passage (91 h after the initial drug addition). In general, high correlation was evident between technical replicates within the same omics dataset (Appendix Fig S1).

### Consistency of community composition across omics measurements

We first evaluated similarities and differences between the omics measurements in their ability to estimate species abundance. For sequencing‐based omics methods, we performed both naïve analyses with commonly used computational pipelines that do not use the information about synthetic community composition (DADA2 for 16S rRNA amplicon sequencing (Callahan *et al*, 2016), mOTUS v2.5 for metagenomics and metatranscriptomics (Milanese *et al*, 2019)), and targeted analyses based on mapping to the 32 reference genomes of species comprising our community (Materials and Methods). Within each omics method, both computational approaches produced highly similar results (Appendix Fig S2). As the composition‐naïve approach only yields genus‐level resolution for 16S rRNA sequencing data (Knight *et al*, 2018), we used the reference genome mapping approach that yields higher resolution for all methods for comparison of community composition across omics types. For consistency, the same methodology (reference genome mapping) was used for metagenomics and metatranscriptomics. For metaproteomics data, we estimated species abundance by summing protein intensities for all proteins assigned to each species and dividing these values by the total protein intensity in each sample, as suggested previously (Kleiner *et al*, 2017).

**Figure 2:**
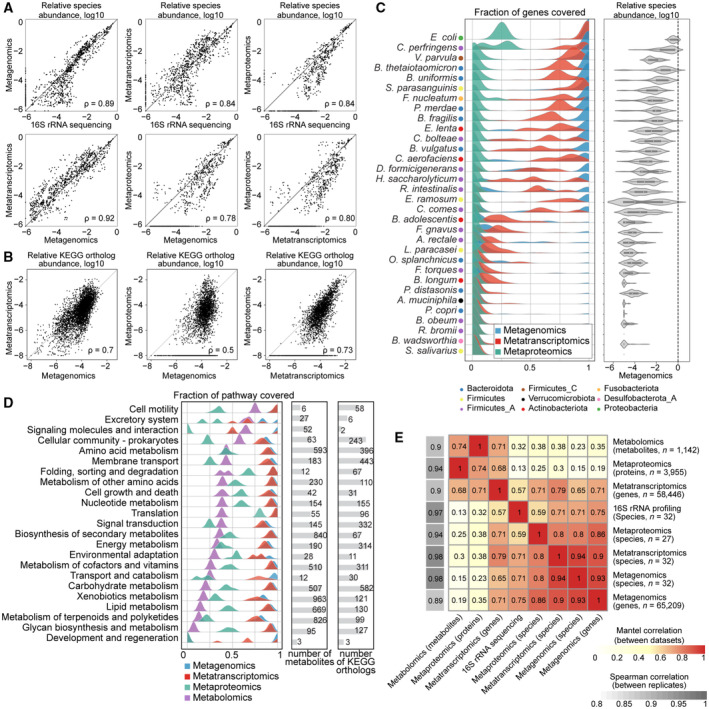
Comparison of species and feature abundances and functional coverage across omics methods Scatter plots representing species abundance defined as relative abundance of corresponding omics measurement in each sample. Each dot represents single species abundance in one sample. ρ, Spearman correlation coefficient.Scatter plots representing gene, transcript or protein abundance linked through KEGG orthology. Each dot represents a single KEGG ortholog in one sample.Left: Genome coverage of each of the omics datasets across samples for each species, right: relative species abundance estimated by metagenomics. The fraction of coverage is defined as the number of genes to which at least one read was mapped (for metagenomics and metatranscriptomics), or the number of detected proteins for metaproteomics divided by the total number of genes in the corresponding genome. (Metagenomics *n* = 75 samples, metatranscriptomics *n* = 101 samples and metaproteomics *n* = 112 samples).KEGG pathway coverage. For the metabolomics dataset, pathway coverage is defined as the number of unique pathway metabolites tentatively detected in at least one sample, divided by the total number of metabolites in the pathway. For metagenomics, metatranscriptomics and metaproteomics, KEGG orthologs are used instead of pathway metabolites.Heatmap of Mantel correlations across omics methods and Spearman correlation between replicates within each omics method.

**Figure EV2:**
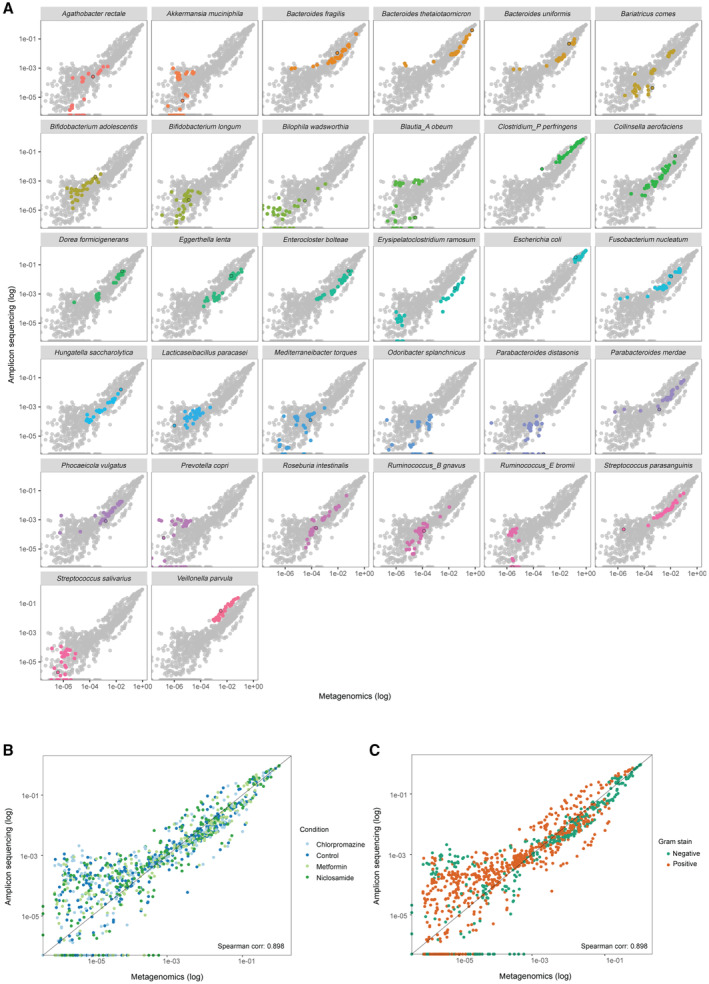
Differences between species abundances estimated by metagenomics and 16S sequencing are not species‐, condition‐ or Gram‐type specific Metagenomics versus 16S sequencing species abundances coloured by species.Metagenomics versus 16S sequencing species abundances coloured by condition.Metagenomics versus 16S sequencing species abundances coloured by Gram staining.

We compared relative species abundances between all pairs of omics methods except for metabolomics, which by nature represents total metabolite measurements in the community and does not allow to separate compounds by species. Based on the correlation analysis, we found the abundance estimates to be highly similar (minimum Spearman correlation coefficient ρ = 0.78). Congruence was more pronounced for highly abundant species (Fig 2A). Specifically, metagenomics and metatranscriptomics were the most similar of all pairwise comparisons (ρ = 0.92). Further, 16S rRNA amplicon sequencing showed high similarity with metagenomics for species with relative abundances higher than 0.001% (ρ = 0.89). However, for several species with low relative abundances, 16S rRNA sequencing provided higher relative abundance estimates compared to metagenomics, while other species, detected by metagenomics, were not detected with 16S rRNA sequencing. For this observation, no clear taxon‐specific or condition‐specific effect was found (Fig EV2A–C), indicating that the differences at these low relative abundances are most likely a result of differences in sequencing depth per sample, as has been previously reported (Pereira‐Marques *et al*, 2019; Durazzi *et al*, 2021). Although metaproteomics is not yet widely used for species abundance estimation, we found the corresponding estimates in good agreement with the other omics methods, but only for species with relative abundance above 1% (ρ = 0.78–0.84; 16 out of 29 species detected across all the samples). This indicates that metaproteomics is less sensitive than sequencing‐based methodologies for species abundance estimation, as has also been observed for *in natura* metaproteomics studies (Zhang & Figeys, 2019). Our results show generally high consistency between omics data types in relative species abundance estimations, and underline that metaproteomics can, in principle, provide robust species abundance estimates, at least for synthetic microbial communities, albeit with lower sensitivity.

### Consistency of functional profiles across omics measurements

For each protein‐coding gene of each species, we can compare relative abundances across the three molecular layers: gene (metagenomics), transcript (metatranscriptomics), and protein (metaproteomics). We performed such pairwise comparisons both for individual genes across all species (Appendix Fig S3) and for genes grouped based on the KEGG orthology (Kanehisa *et al*, 2017; Fig 2B). The correlation between metagenomic and metaproteomic estimates of gene and protein abundances was moderate (ρ = 0.5 for KEGG grouped features and ρ = 0.48 for all non‐zero genes and proteins). Metatranscriptomics and metaproteomics were the most similar (ρ = 0.73 for KEGG orthologs and ρ = 0.60 for transcripts and proteins), followed by metagenomics and metatranscriptomics (ρ = 0.7 for KEGG orthologs and ρ = 0.61 for genes and transcripts).

To systematically assess how much information on the functional level is captured by metagenomics, metatranscriptomics and metaproteomics for different species, we estimated gene and pathway coverage by calculating the proportion of genes or pathways that were detected by each method (Fig 2C and D). We found that 18 out of 32 species had an almost complete coverage (> 90%) in metagenomics, indicating that for these species most of the genes were recovered in all samples measured in this experiment (Fig 2C; in total 101,559 out of 103,921 possible protein‐coding genes were detected at least once in the metagenomics dataset). This was not the case for 14 low‐abundant species, for which the average gene content coverage was < 20%. For metatranscriptomics, the coverage was generally lower than for metagenomics (91,094 out of 103,921 possible transcripts detected at least once). This is however expected as not all genes are expressed in any given condition. Metaproteomics coverage was found to be much lower than metagenomics and metatranscriptomics (9,144 out of 103,921 predicted proteins). This may be due to the limited dynamic range: In contrast to mass‐spectrometry‐based measurements, sequencing‐based methods include an amplification step that increases the amount of material and makes it possible to cover rare transcripts and genes. For *Escherichia coli*, the most abundant species in our synthetic community, the maximum coverage of proteins across all samples did not exceed 30% (1,428 proteins out of 4,978 [29%] predicted proteins compared to 4,978 genes out of 4,978 predicted genes [100%] for metagenomics and 4,962 transcripts out of 4,978 transcripts [99%] for metatranscriptomics). This result is lower than state‐of‐the‐art single species proteomics experiments, where around ~ 62% (2,586 detected proteins out of 4,189 predicted proteins) of bacterial proteins are captured (Mateus *et al*, 2020), likely due to the increased sample complexity in the community context, the increased search space of proteins and the presence of highly similar sequences in homologous proteins (where peptides cannot be unambiguously mapped to one protein).

Since metabolomics data reflect the total pools of metabolites in the sample and cannot be analysed at the species level, we assessed the coverage of metabolic pathways defined in the KEGG database and compared it to pathway coverages by other omics methods (Fig 2D). For our analysis, we used 1,142 detected ions tentatively annotated as 3,488 possible metabolites by matching their accurate masses against the HMDB database (Wishart *et al*, 2018). This approach generally provides only low confidence in individual annotations and is unable to distinguish between isomers, yet ensures very broad tentative metabolome coverage. For metabolic pathways annotated in bacterial genomes, we observed an average pathway coverage of 35% for metabolomics, as compared to 44% for metaproteomics and 86% for metatranscriptomics. Even though direct comparison of omics methods is challenging, we believe that the lower coverage for metabolomics has several explanations. First, we measured metabolites in supernatant samples, to capture the drug and its metabolites and the secreted metabolites that play important roles in microbial communities in cross‐feeding and signalling (Yu *et al*, 2022). A more in‐depth study could also additionally use the cell pellet for metabolomics, for example, to detect bioaccumulation (Klünemann *et al*, 2021). This means that components of the rich medium masked part of the signal (e.g., amino acids, peptides and polysaccharides), and extracellular products of bacterial metabolism, especially produced by only one or few species, may therefore be too dilute to be detected. Second, only a subset of all metabolites present in the bacterial cell will be secreted outside of the cell. Third, to calculate metabolic pathway coverage, we assumed that each pathway consists of metabolites that are produced or consumed by metabolic enzymes annotated in bacterial genomes, which is likely an overestimation of pathway sizes, since presence of an enzyme‐coding gene in the genome does not necessarily imply that this enzyme was expressed or that its reactants were present in our experimental conditions.

To further compare the samples measured with different omics methods, we performed a Mantel test, which measures a correlation coefficient between sample similarity matrices calculated based on each omics data type individually (Fig 2E). For example, while it is not possible to directly compare matrices of species and protein abundances, it is possible to calculate sample similarity matrices for these two methods that can then be compared with each other. Notably, transcript abundance as measured by metatranscriptomics showed a high correlation (≥ 0.57) with sample distance matrices of all other omics measurements, underlining that this method captured both species abundance and functional information in our experiment. Hierarchical clustering of Mantel correlation coefficients revealed two groups, which shared transcript abundance data from metatranscriptomics as a common member: one group with species abundance data (from metagenomics, metatranscriptomics and metaproteomics) and gene abundance (metagenomics); and the second group with protein abundance data (metaproteomics) and metabolite abundances. The emergence of these groups can be explained by the nature of the data used to calculate sample distance matrices: species and gene abundances in one group, and functional feature abundances in the other group. Altogether, metatranscriptomics was found to be the most universal and versatile readout, as it can both provide robust and sensitive estimates of species abundance, and at the same time reflects functional changes, which are in concordance with protein changes detected by metaproteomics.

### Chlorpromazine treatment strongly affects community composition

**Figure 3:**
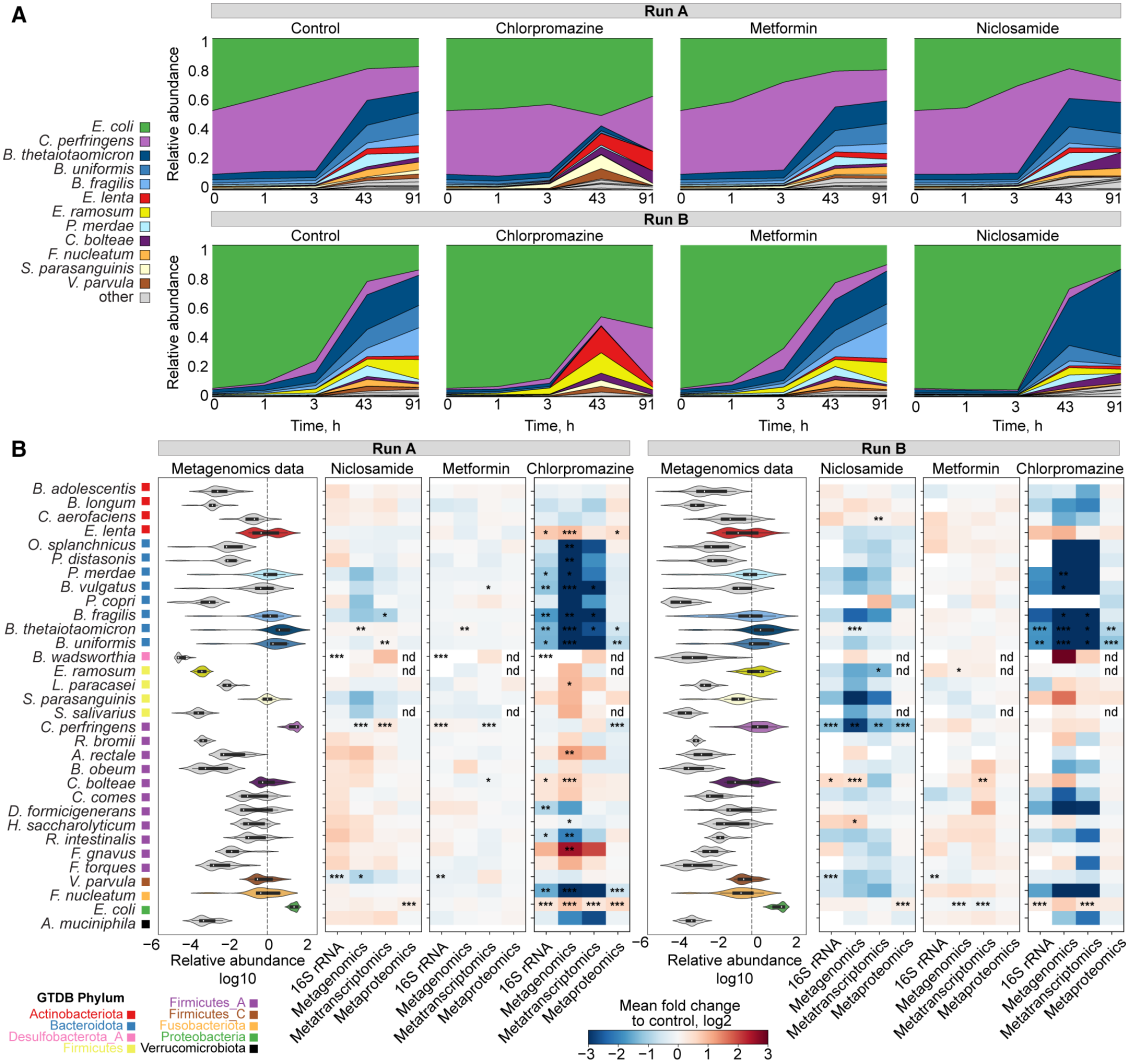
Changes in community composition upon drug perturbation Relative species abundance changes over time in the three drug conditions and control. Time 0 indicates timepoint of the drug addition 5 h after the passage in the fresh medium. Relative abundance measured from metagenomics data.Left, distribution of relative species abundance for each species across all samples (all conditions and timepoints). Right, heatmap of species abundance fold changes measured by different omics methods for each drug condition versus control. Significance of changes estimated by the ANCOM test is indicated by asterisks: *changes detected at 0.7 threshold of W statistic; **changes detected at 0.8 threshold; ***changes detected at 0.9 threshold; nd, not detected.

**Figure EV3:**
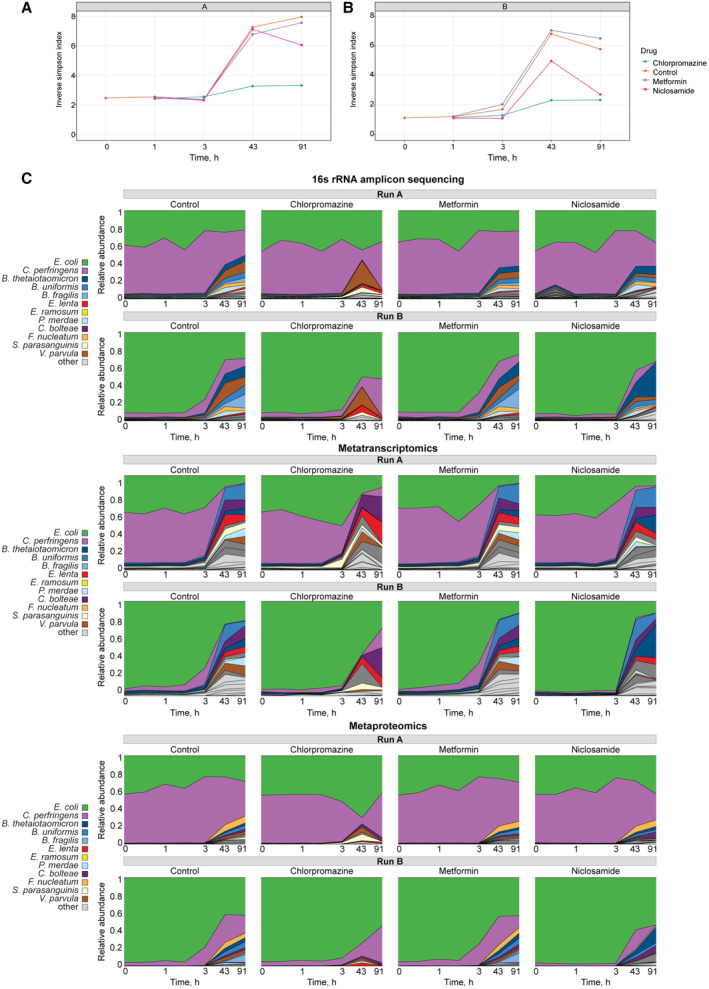
Chlorpromazine strongly affects community composition A, BCommunity alpha diversity measurements over time after drug treatment for runs A and B, correspondingly.CRelative species abundance changes over time in the three drug conditions and control. Relative abundance measured from 16S rRNA amplicon sequencing, metatranscriptomic and metaproteomic data.

After testing the technical consistency between omics measurements in a synthetic microbial community, we explored the impact of drug perturbations on the community composition and the respective responses at species, gene, transcript, protein and metabolite levels. For the control condition and all perturbations (chlorpromazine, metformin and niclosamide), similar dynamic changes in alpha diversity were observed over time. In general, the alpha diversity (inverse Simpson index) increased as the community grew over time after inoculation, however, this increase was lower for chlorpromazine compared with the other drugs and the control condition (Fig EV3A and B). We observed different community dynamics between runs A and B during the exponential phase: *E. coli* and *C. perfringens* were the most abundant species in all conditions in run A (Figs 3A and EV3C), while *E. coli* dominated community composition during exponential phase in run B. However, community compositions became more similar between the runs at 43 h after drug treatment (Appendix Fig S4). These analyses revealed that the addition of metformin and niclosamide had negligible effects on the community composition, while chlorpromazine treatment shifted the community composition in both runs.

To identify differentially abundant species after drug perturbation, we analysed the composition of microbiomes by comparing species abundances in drug‐treated samples against control samples estimated by each omics type (Fig 3B; ANCOM (Mandal *et al*, 2015)). This analysis revealed that most members of the Bacteroidota phylum (*Odoribacter splanchnicus*, *Parabacteroides distasonis*, *Phocaeicola vulgatus*, *Bacteroides fragilis*, *Bacteroides thetaiotaomicron* and *Bacteroides uniformis*) were less abundant in chlorpromazine‐treated samples. This reduction in Bacteroidota abundance was detected across all four omics methods capturing community composition, indicating that each of these methods is capable of detecting strong signals of species abundance change. In addition to Bacteroidota, *Fusobacterium nucleatum* was found to be less abundant in chlorpromazine‐treated samples. In contrast, the other two drugs did not cause major shifts in relative abundances: although ANCOM test identified significant changes of abundance of several species, their relative abundance was not changing more than two‐fold (Fig 3B). In summary, we found a consistent and substantial depletion of species belonging to the phylum Bacteroidota upon chlorpromazine treatment.

### Multi‐omics measurements capture functional response of the community to all three drugs

**Figure 4:**
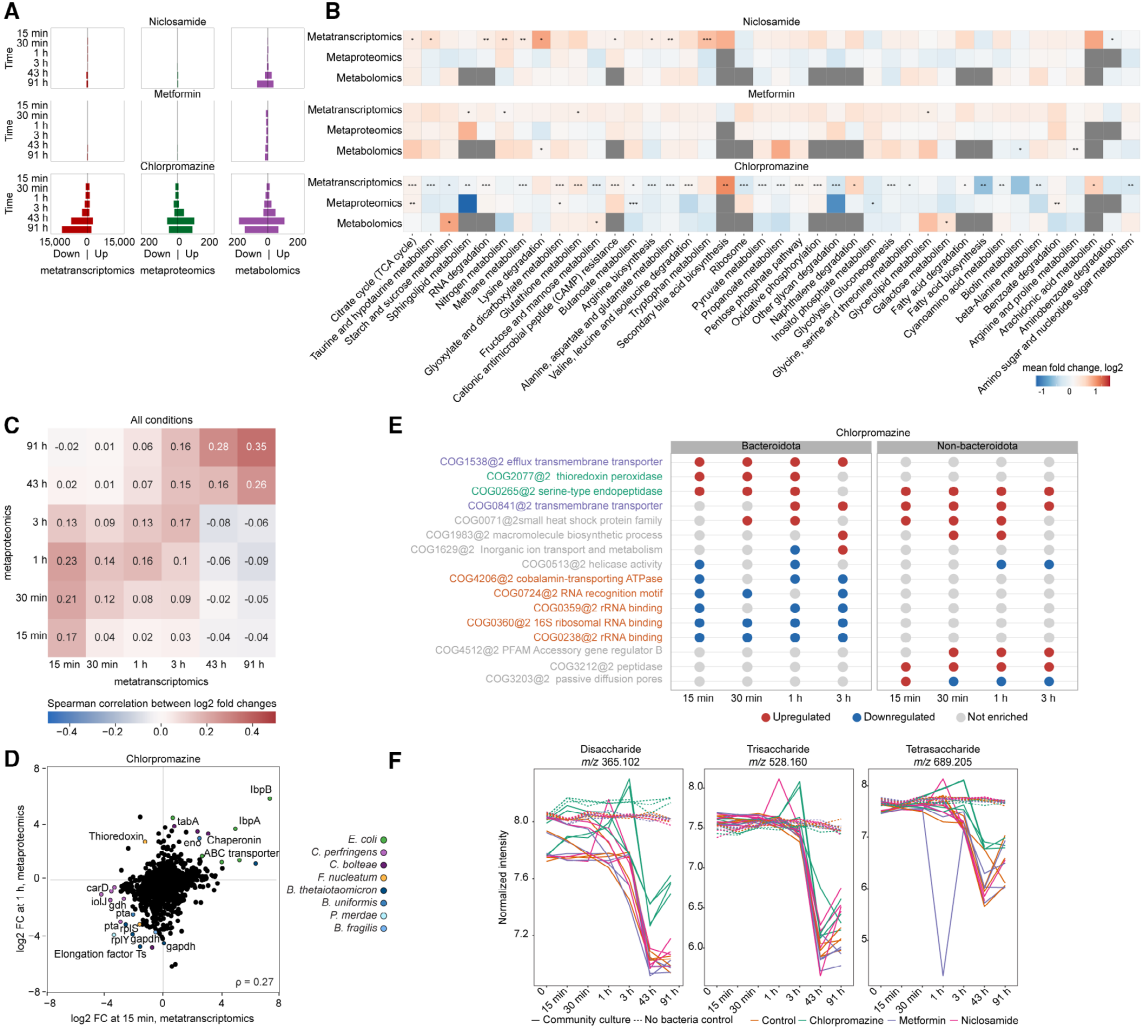
Functional analysis of transcript, protein and metabolite response after niclosamide, metformin or chlorpromazine treatment Number of differentially abundant transcripts, proteins and metabolites.Pathway enrichment analysis across all conditions and time points. *P*‐values are indicated by asterisks: *P ≤ 0.05, **P ≤ 0.01, ***P ≤ 0.001.Heatmap representing Spearman correlation between fold changes (relative to control) detected by metatranscriptomics and metaproteomics across all drug perturbations.Scatterplot depicting protein fold changes (relative to control) detected after 15 min of chlorpromazine exposure by metatranscriptomics versus after 1 h of exposure by metaproteomics.COG enrichment analysis differentiating between species susceptible to chlorpromazine treatment (Bacteroidota) and non‐susceptible species (non‐Bacteroidota). COGs that are enriched in upregulated genes are coloured in red, while COGs that are enriched in downregulated genes are coloured in blue. Only COGs that were found to be significantly enriched in at least three out of four early time points are shown. COG names that are coloured are discussed in more detail in the main text.Di‐, tri‐ and tetra‐saccharide abundances as measured by untargeted metabolomics (tentative metabolite annotation is based on m/z values indicated in the panel titles). The lines are coloured according to the experimental conditions (chlorpromazine, metformin, niclosamide and control), and the line type represents whether these are community culture or non‐bacterial controls.

As compositional shifts do not provide information on the mechanisms of response of each community member, we investigated these functional responses in more detail by performing differential analysis of metatranscriptomic, metaproteomic and metabolomic datasets after a normalisation step wherein taxonomic abundance effects were reduced (see ‘Gene, transcript and protein counting’ in the Materials and Methods section). The highest number of differentially abundant transcripts, proteins and metabolites were found in samples treated with chlorpromazine (adjusted *P*‐value < 0.001 and absolute fold change > 4 compared with the control for metatranscriptomics, adjusted *P*‐value < 0.05 and absolute fold change > 1.5 for metaproteomics and metabolomics; Fig 4A), which is in line with our findings that chlorpromazine caused the largest disruption to bacterial community (Fig 3B). Transcriptional response to chlorpromazine is detected already after 15 min of treatment across species belonging to different phyla, suggesting that, although Bacteroidota show the strongest response, other species also adapt their gene expression.

In order to evaluate similarities between functional responses across omics data types, we performed pathway enrichment analysis of differentially abundant features between drug treatment and controls across all time points using the KEGG pathway annotations (Fig 4B). In general, we detected less overlap between omics layers on the functional level compared to species abundance analysis, as no single pathway was statistically significant in the enrichment analysis of all three functional omics datasets. Across all conditions, five pathways were found to be significantly enriched upon drug treatment compared to the control condition in two omics data types, while 35 pathways were statistically significantly enriched in only one omics dataset. The largest number of significantly enriched pathways was found in chlorpromazine‐treated samples for metatranscriptomics data.

For our metformin‐treated samples, we did not observe substantial effects of metformin neither on community composition nor on transcript or protein abundance in our study, at least at the concentrations used. Only a small number of pathways were significantly overrepresented (pFDR < 0.001 for metatranscriptomics and pFDR < 0.05 for metaproteomics and metabolomics) within the set of up‐ and downregulated features (transcripts/proteins/metabolites) in metformin‐treated samples (Fig 4B). Further inspection of putative metabolites involved in these pathways showed that their abundance also decreased upon addition of metformin in the non‐bacterial control samples (Appendix Fig S5). This indicates that metformin primarily interferes with the measurement of these putative metabolites, probably due to their chemical similarity, underlining the importance of including non‐bacterial control samples to study drug response. However, we cannot exclude that metformin also interacts with lysine and arginine metabolism pathways in bacteria, as reported before (Forslund *et al*, 2015; Pryor *et al*, 2019). In the previous single‐strain experiments, metformin at the same concentration was shown to have an effect on several of the tested species (Maier *et al*, 2018). We attribute this discrepancy to the possibility that these species show a different behaviour in a community setting compared to a single culture setting, as already shown for other species (D'hoe *et al*, 2018). Unfortunately, our datasets do not provide any further hypotheses as to what the underlying cause of this protective community effect could be.

**Figure EV4:**
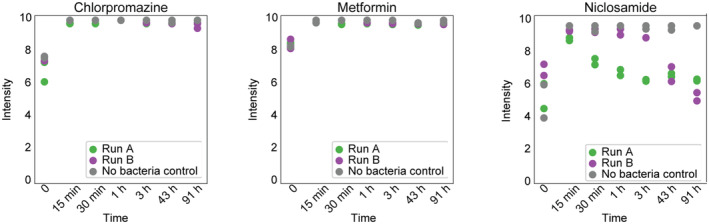
Drug profiles measured over time Drug concentrations were measured both during the community experiments and in controls in sterile medium.

For niclosamide‐treated samples, 10 pathways were significantly enriched (pFDR < 0.001) among regulated transcripts, including amino acid and nitrogen metabolism. Transcripts of nitrogen metabolism pathway upregulated in the early time points (15 min, 30 min, 1 h, 3 h) were annotated as NAD‐specific glutamate dehydrogenase (belonging to the Cluster of Orthologous Groups COG0334 from the EggNOG database present in *B. thetaiotaomicron*, *P. vulgatus*, *B. fragilis*), hydroxylamine reductase (COG1151 in *C. perfringens*, *B. uniformis*) and carbamate kinase (COG0549 in *Eggerthella lenta*) (Appendix Fig S6). Previously, NAD‐specific glutamate dehydrogenase was found to be upregulated in response to nitrogen availability in *Mycobacterium smegmatis*, where it is assumed to have a de‐aminating activity (Harper *et al*, 2010). Furthermore, hydroxylamine reductase and carbamate kinase are enzymes belonging to the family of oxidoreductases which both act on nitrogenous compounds. Therefore, the upregulated pathway and its transcripts suggest increased nitrogen metabolism in niclosamide‐treated samples. Further examination of our metabolomic dataset revealed that niclosamide gets degraded in both runs of the experiment (Fig EV4), which could explain the observed absence of perturbations of the community composition. Nitroreductases are known to detoxify niclosamide (Copp *et al*, 2020). While members of the nitroreductases family (COG0778) are expressed, we did not observe significant changes in their expression levels upon treatment with niclosamide. Additional follow‐up experiments are needed to elucidate the mechanisms underlying the microbial degradation of niclosamide and the roles of individual community members.

### Chlorpromazine induces stress response and metabolic changes in the community

Since the number of differentially abundant features and pathways was high in chlorpromazine‐treated samples (Fig 4A and B), we tested whether there are features that change concordantly across omics layers. We first compared transcript and protein fold changes upon perturbation, which revealed general agreement between relative changes in gene expression and protein abundance, with transcript fold changes at each time point correlating more strongly with protein changes at later time points (Fig 4C; Appendix Fig S7), likely reflecting the delay between transcription and translation processes. Based on this analysis, we assessed the most prominent and concordant changes between metatranscriptomics and metaproteomics 15 min and 1 h after chlorpromazine addition, respectively (Fig 4D). The most concordantly downregulated features were proteins and genes of Bacteroidota species and *F. nucleatum*, including ribosomal proteins, elongation factors, and central carbon metabolism enzymes *gldA* (glycerol dehydrogenase), *gapdh* (glyceraldehyde 3‐phosphate dehydrogenase), and *pta* (phosphate acetyltransferase), the latter two being downregulated in several species (Fig 4D). Furthermore, the most upregulated features found both in metatranscriptomics and metaproteomics were stress response genes in *E. coli*, such as the small heat shock proteins IbpA and IbpB (Inclusion body‐associated proteins A and B), other chaperones, and ABC transporters. IbpA and IbpB serve as a first line of defence against protein aggregation (Miwa *et al*, 2021). In addition to *ibpA* and *ibpB*, we found upregulation of the transcriptional regulator *rpoH* and the chaperones *dnaK* and *groEL*, which are also involved in heat shock response (Yura, 2019; Appendix Fig S8). Together, these results show that chlorpromazine causes the activation of a stress response in *E. coli*, probably due to induction of protein aggregation either directly or indirectly.

**Figure EV5:**
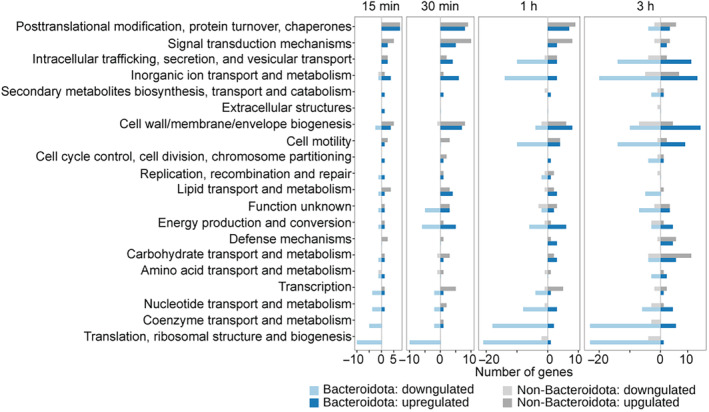
Number of protein‐coding genes grouped by COG category changing per time point upon chlorpromazine treatment Bacteroidota species quickly downregulated genes involved in translation and the ribosome compared to other species.

We then tested whether genes associated with stress response were differently regulated between chlorpromazine‐susceptible and non‐susceptible species. Two COGs related to the stress response were enriched in upregulated genes in at least two of the four early time points in the depleted (susceptible) species (Fig 4E, annotated in green, Fig EV5). One of them, COG0265, is upregulated by both susceptible and non‐susceptible species and encompasses serine proteases (e.g., HtrA proteins such as DegP and DegQ), which represent an important class of chaperones and heat‐shock‐induced serine proteases, protecting periplasmic proteins. Furthermore, two COGs enriched in upregulated genes were related to (multidrug) transporter activity. COG1538, which contains genes annotated as membrane protein OprM, was the only COG enriched in upregulated genes by Bacteroidota in all four early time points (Fig 4F, annotated in purple). In *Pseudomonas aeruginosa*, OprM is part of MexAB–OprM, a multidrug efflux pump of the resistance‐nodulation‐cell division (RND) superfamily, where it plays a central role in multidrug resistance by transporting drugs from the cytoplasm across the inner and outer membranes outside the cell envelope (Alekshun & Levy, 2007; Tsutsumi *et al*, 2019). RND‐efflux pumps are found in a number of Gram‐negative bacteria, for example, AcrAB–TolC is found in *E. coli* (Du *et al*, 2018) while *Bacteroides fragilis* harbours multiple copies of RND pumps BmeABC (Ghotaslou *et al*, 2018). Further, in addition to COG1538 (OprM homologues), also COG0841 containing homologues of the MexB/AcrB/BmeB protein (Fig 4E, also annotated in purple) was found to be enriched in upregulated genes, both in Bacteroidota and non‐Bacteroidota species. These observations suggest an important role of the AcrAB–TolC/MexAB‐OprM/BmeABC efflux pumps in determining chlorpromazine susceptibility. Indeed, a recent study showed that chlorpromazine is both a substrate and an inhibitor of the AcrB multidrug efflux pump in *Salmonella enterica* and *E. coli* (Grimsey *et al*, 2020). Together, our results suggest that chlorpromazine could also be an inhibitor of BmeB, the AcrB/MexB homologue in Bacteroidota species and that this, potentially in combination with protein aggregation, could be one of the reasons explaining why Bacteroidota are affected by chlorpromazine treatment.

Finally, depletion of Bacteroidota and downregulation of their genes involved in saccharide uptake might explain the enrichment of ‘Starch and sucrose’ and ‘Fructose and mannose metabolism’ pathways among metabolites more abundant upon chlorpromazine treatment compared to the control samples (Fig 4B). As *Bacteroides* species are known to be capable of metabolising a wide variety of polysaccharides (Schwalm & Groisman, 2017), we believe that the higher abundance of ions tentatively annotated as oligosaccharides after chlorpromazine treatment (Fig 4F) measured by metabolomics is a result of their reduced consumption by these species. At the same time, the abundance of Bacteroidota is decreased compared to control. With the present data, we are not able to quantify the relative contributions of reduced Bacteroidota biomass and lower metabolic activity per cell to the observed differences in metabolite levels.

Taken together, by integrating multi‐omics measurements, we propose that a series of events happens upon treatment with chlorpromazine: (i) a stress response is induced across several bacterial species with overexpression of *ibpA* and *ipbB* chaperones being the most pronounced response in *E. coli*; (ii) this stress response involves upregulation of AcrB/BmeB type of RND pumps, which may be bound and blocked by chlorpromazine in a species‐specific manner; (iii) Bacteroidota species are more susceptible to chlorpromazine and are quickly depleted from the community while also downregulating genes involved in saccharide uptake, both of which result in (iv) higher levels of oligosaccharides in the culture medium due to the reduced ability of the perturbed community to utilise them.

## Discussion

In this study, we evaluated the impact of drug perturbations on a synthetic gut microbial community by analysing five different omics data types in a highly controlled *in vitro* experiment. In general, we found concordance between all omics data types regarding the estimation of community composition (taxonomic profiling). To our knowledge, this is the first study to systematically compare taxonomic profiles obtained by four omics data types and can thus serve as a baseline for integrating different data types in ‘in natura’ settings. Using the synthetic community, we could show a high correlation between metagenomics and metatranscriptomics (ρ = 0.92), similar to a previous study that used only these two omics methods (ρ = 0.81; Heintz‐Buschart & Wilmes, 2018). The taxonomic profiles obtained from our metaproteomics dataset, which is increasingly used in microbiome studies (e.g. Kleiner *et al*, 2017; Kleikamp *et al*, 2021), showed correlations between ρ = 0.78 and ρ = 0.84 with all other omics for species with a relative abundance higher than 1%. Although the number of detected proteins and the detection limits remain to be improved, we showed that species abundance estimates can be derived from metaproteomics in a relatively simple, defined microbial community.

For a defined community as used here, 16S amplicon sequencing would be sufficient to capture species abundance. However, when closely related species are used or natural communities are cultured *ex vivo*, a method based on shotgun sequencing is necessary. If the available resources make it necessary to prioritise, then metatranscriptomics sequencing would be preferred, as its estimation of species abundance is highly correlated with metagenomics, but it additionally allows for functional profiling and offers insights into alterations in gene expression.

Metabolomics measurements offer a complementary readout of the community functions. In our experiment, metabolomics revealed differences in the degradation of the studied drugs and suggested the links between the abundance of oligosaccharide compounds in the culture medium and changes in Bacteroidota abundance in response to chlorpromazine treatment. However, since our flow injection approach enabled acquisition of only exact ion masses (MS1), our dataset contains many ambiguous ion annotations. Follow up studies collecting tandem mass spectra (MS1 and MS2) and using chemical standards are required to confirm the identities of the changing metabolites (Schymanski *et al*, 2014). Furthermore, our dataset contains only extracellular metabolite measurements, and thus only provides indirect information on intracellular functional changes in the bacterial community. Of the three drugs used for perturbation, only chlorpromazine caused a large disturbance in the community composition. Surprisingly, metformin, which has been shown to alter the gut microbiome in patients (Forslund *et al*, 2015; Wu *et al*, 2017), did not perturb the community in our study, even though our earlier study suggested that the growth of at least four different species is inhibited by metformin at the concentration used in monocultures (*F. nucleatum*, *B. longum*, *P. copri* and *P. merdae*; Maier *et al*, 2018). This observation hints at a protective effect from the community, although this protective effect is not caused by drug degradation, as metformin concentrations remained high during the course of experiment (Fig EV4), but could be due to other interactions between members of the bacterial community, for example, by altered growth rates (D'hoe *et al*, 2018). Similarly, niclosamide was expected to cause a depletion of most of the members of the synthetic community, except for *E. coli* and *B. wadsworthia* (Maier *et al*, 2018), which was not observed in this study, also pointing to community‐related protection effects. Our metatranscriptomic data revealed an upregulation of genes related to nitrogen metabolism, while niclosamide concentration decreased during incubation, which was not observed in the non‐bacterial controls. Therefore, we believe that certain species are capable of degrading niclosamide, which ultimately protected the whole community against possible inhibitory effects of niclosamide treatment.

For chlorpromazine, the observed depletion of Bacteroidota species was in concordance with single species experiments (Maier *et al*, 2018). The antibiotic activity of chlorpromazine was reported relatively soon after its first usage in the 1950s (Kristiansen & Vergmann, 1986; Dinan & Cryan, 2018). Its antibiotic mechanism of action is described to be multifold and includes effects on the cell membrane, energy generation and interference with cell replication due to DNA intercalation in *E. coli* (Grimsey *et al*, 2020). In our study, several genes and proteins related to protein aggregation were found to be upregulated in the metatranscriptomic and metaproteomic data in *E. coli* and other community members. One study already reported protein aggregation of bovine insulin after chlorpromazine treatment (Bhattacharyya & Das, 2001). However, it remains unclear whether chlorpromazine can cause protein aggregation in microbes either directly or indirectly, a hypothesis that should be followed‐up in future experiments.

Finally, we identified upregulation of RND‐type efflux pumps in the Gram‐negative bacteria, even in the Bacteroidota species that were severely depleted. It was recently shown that in *S. enterica* and *E. coli*, chlorpromazine is both a substrate and an inhibitor of AcrB, the inner membrane transporter of the tripartite system AcrAB–TolC, which is an RND‐type efflux pump (Bailey *et al*, 2008a; Grimsey *et al*, 2020). Based on our data, we hypothesise that BmeB, the AcrB homologue in Bacteroidota, is also susceptible to chlorpromazine inhibition as we found upregulation of this and related genes, similar to what has been described by others in single species experiments (Grimsey *et al*, 2020). The suggested mechanism could be of significance in the battle against the rising multidrug resistance of *Bacteroides fragilis*, a commensal bacterium that can act as a virulent pathogen when it escapes its normal niche (Wexler, 2007, 2012; Niestępski *et al*, 2019). However, chlorpromazine's antimicrobial activity generally occurs at concentrations higher than those clinically achievable (Grimsey & Piddock, 2019). Therefore, it is possible that, similarly as suggested for *S. enterica*, chlorpromazine could act as an antimicrobial adjuvant for Bacteroidota where its inhibition of RND‐type efflux pumps prevents the extrusion of administered antibiotics (Grimsey *et al*, 2020). From the perspective of human health, these results underline the detrimental effect of antipsychotics on the gut microbiome reported before (Dinan & Cryan, 2018). However, the revealed phylum‐specific differences provide an opportunity to explore whether complementation of antipsychotic therapy with Bacteroidota‐promoting dietary interventions could improve mental health and increase patients' quality‐of‐life by restoring a healthy microbiota (Patnode *et al*, 2019).

In conclusion, we directly compared data from multiple omics methods and showed that they agree on species abundance estimation of a defined and drug‐perturbed microbial community *in vitro*. Those methods that are able to detect functional information also correlate with each other, albeit to a lower degree. We could also confirm expected time delays between transcriptional and translational responses to perturbations, underlining that these methods reveal biological insights that happen at different time scales. While we were not able to detect the induction of metabolising enzymes in response to drug perturbation, we could detect the upregulation of other resistance mechanisms such as transporters just 15 min after the perturbation (Fig 4). Future studies could therefore investigate a broader panel of drugs with the timepoints established in this study. Although multi‐omics analysis of natural communities is hampered by their increasing complexity, combining multiple omics measurements allows to measure the response of the community to perturbations across molecular layers and provides information that is not achievable by any method alone.

## Materials and Methods

### Species and drug selection

The species used in this study represent a subset of abundant and prevalent species from the human gut. In total, 32 species were selected based on our previous work (Maier *et al*, 2018; Tramontano *et al*, 2018). The bacterial isolates were received from DSMZ, BEI Resources or ATCC and Dupont Health & Nutrition. The drugs were chosen because of their antimicrobial activity (Maier *et al*, 2018) and diversity in therapeutic usage.

### Reference genomes

Reference genomes were downloaded from RefSeq in March 2019 (release 92) and reannotated using Prokka v1.14.0 (Seemann, 2014). Taxonomic classification was based on GTDB taxonomy release 95 (Parks *et al*, 2018) and inferred using GTDB‐Tk v1.3.0 (Hyatt *et al*, 2010; Matsen *et al*, 2010; Price *et al*, 2010; Eddy, 2011; Ondov *et al*, 2016; Jain *et al*, 2018; Chaumeil *et al*, 2020). Further functional annotations (e.g., the KEGG orthology and eggNOG orthologous group) were retrieved using eggNOG‐mapper v2.0.1 which is based on eggNOG v5.0 (Huerta‐Cepas *et al*, 2017). A cladogram was built by pruning the species cladogram from GTDB (bac120.tree, release 95) using the ETE toolkit (Huerta‐Cepas *et al*, 2016).

### Medium and drug preparation

mGAM medium was prepared according to manufacturer's instructions (HyServe GmbH & Co.KG, Germany, produced by Nissui Pharmaceuticals) and all the single species were grown in this medium except *V. parvula* (Todd‐Hewitt Broth (Sigma‐Aldrich) + 0.6% sodium lactate) and *B. wadsworthia* (mGAM + 60 mM sodium formate + 10 mM taurine). All media were placed in anaerobic chamber 1 day before use under anoxic conditions (Coy Laboratory Products Inc.) (2% H_2_, 12% CO_2_, rest N_2_). Chlorpromazine (TCI Chemicals) and niclosamide (Santa Cruz Biotechnology) were added from DMSO stock solution. Metformin (Sigma) was added as powder directly into the medium after which the medium was filter‐sterilised. Final concentrations of each drug were chosen based on previous work (Maier *et al*, 2018) with concentrations of 5 mM for metformin and 20 μM for chlorpromazine and niclosamide. The higher concentration for metformin is motivated by previously published data, which showed that a concentration of 20 μM was not sufficient to impair growth of gut microbiome members *in vitro* (Maier *et al*, 2018; Fig EV4).

### Experimental set‐up and sample collection

Species were pre‐inoculated in isolation on liquid mGAM medium from pure stocks and incubated at 37°C under anaerobic conditions for a period of 3 or 5 days, depending on the growth rate of each species (see Fig 1). The monocultures were subsequently mixed in equal proportions based on their OD and then inoculated in 100 ml of mGAM liquid medium. To allow species to reach a stable state (stabilisation phase), the mixed culture was grown for 48 h after which 1 ml was transferred to fresh medium. In total, three passages were performed and after the second transfer OD measurements were taken to determine the start of the exponential phase.

Following the stabilisation phase, the mixed community was inoculated in medium prepared with one single drug or DMSO (control) as soon as the community reached the exponential phase (OD roughly equal to 2–3). The cultures were subsequently sampled (3 mL) at fixed time intervals (0 min, 15 min, 30 min, 1 h, 3 h, 48 h), transferred to fresh medium (with drugs or DMSO) after 48 h and then sampled again 48 h later (or 96 h after the start of the experiment). The whole experiment was performed twice (labelled as run A and run B).

1.5 ml of each collected sample was centrifuged (30 s at max speed) after which the supernatant was removed and the cell pellet was stored at −80°C until further processing for DNA and RNA extractions. For protein and metabolite extraction, again 1 ml of each collected sample was centrifuged (30 s at max speed) and 450 μl of supernatant was used for metabolite extraction while the cell pellet was used for protein extraction (proteins in the cells). The remainder of the samples was frozen at −80°C as backup.

### DNA and RNA extraction

Genomic DNA and total RNA were extracted from the same flash‐frozen samples using Allprep Powerfecal DNA/RNA kit (Qiagen, Hilden Germany) following the manufacturer's protocol but an additional phenol–chloroform extraction step of 700 μl was performed after lysis. DNA yield was measured by using Qubit™ dsDNA HS Assay Kit (Qubit, Waltham, Massachusetts, USA), split into two aliquots for ribosomal 16S rRNA amplicon sequencing and metagenomic shotgun sequencing and was stored at −20°C. RNA yield was measured via Bioanalyzer (Agilent, Santa Clara, California, USA) with Pico and Nano chips depending on the sample concentration and stored at −80°C for further analysis.

### 16S rRNA amplicon, metagenomic and metatranscriptomic sequencing

For 16S rRNA amplicon sequencing, extracted DNA was amplified using primers targeting the V4 region of the 16S rRNA gene on the F515 and R806 primer pair (Caporaso *et al*, 2011). PCR was performed according to the manufacturer's instructions of the KAPA HiFi HotStart PCR Kits (Roche, Basel Switzerland) using barcoded primers and a two‐step PCR protocol (NEXTflex™ 16S V4 Amplicon‐Seq Kit, Bioo Scientific, Austin, Texas, USA). PCR products were pooled and purified using size‐selective SPRIselect magnetic beads (0.8 left‐sized, Beckman Coulter, Brea, CA, USA). The library was then diluted to 6 pM for sequencing. The library was sequenced on an Illumina (San Diego, USA) MiSeq platform using 2 × 250 bp paired‐end reads at Genomics Core Facility (European Molecular Biology Laboratory [EMBL], Heidelberg, Germany).

Metagenomic libraries for all samples were prepared using the NEB Ultra II and SPRI HD kits with a targeted insert size of 350, and sequenced on an Illumina HiSeq 4000 platform (Illumina, San Diego, CA, USA) in 2 × 150 bp paired‐end with the aim of 1.5 Gbp average setup at the Genomics Core Facility (EMBL, Heidelberg, Germany).

RNA samples were depleted for ribosomal RNA using the NEBNext Bacteria rRNA Depletion Kit (New England Biolabs, Ipswich, Massachusetts, USA). Samples were pooled into a library using the NEBNext Ultra II Directional RNA Library Prep Kit (New England Biolabs) and subsequently sequenced on Illumina NextSeq500 platform (75 bp; single end) at Genomics Core Facility (EMBL, Heidelberg, Germany).

Quality control of raw reads was performed using NGLess (Coelho *et al*, 2019). For metagenomics, reads were trimmed to the longest subread where each base had a Phred score of at least 25. For metatranscriptomics, a sliding window approach was used and reads were trimmed to the longest subread with an average Phred score of 20 (window size: 4 bp). Resulting reads shorter than 45 bp were discarded. To remove possible human contamination, all reads were mapped against a human reference database (release GRCh38.p10, Ensembl; Zerbino *et al*, 2018) using NGLess and samtools (Li *et al*, 2009). Reads with an identity threshold ≥ 90% were discarded. For metatranscriptomics specifically, rRNA reads were also removed from the dataset using SortMeRNA (Kopylova *et al*, 2012) with default parameters.

### Protein extraction

Sample preparation, including protein extraction, digestion and peptide purification was performed according to the in‐StageTip protocol (Kulak *et al*, 2014, 20) with automation on an Agilent Bravo liquid handling platform according to (Geyer *et al*, 2016). In brief, samples were incubated in the PreOmics lysis buffer (P.O. 00001, PreOmics GmbH) for reduction of disulfide bridges, cysteine alkylation and protein denaturation at 95°C for 10 min. Samples were sonicated using a Bioruptor Plus from Diagenode (15 cycles of 30 s). The protein concentration was measured using a tryptophan assay. In total, 200 μg protein of each organism were further processed on the Agilent Bravo liquid handling platform by adding trypsin and LysC (1:100 ratio—μg of enzyme to μg of sample protein). Digestion was performed at 37°C for 4 h.

The peptides were purified in consecutive steps according to the PreOmics iST protocol (www.preomics.com). After elution from the solid phase extraction material, the peptides were completely dried using a SpeedVac centrifuge at 60°C (Eppendorf, Concentrator plus). Peptides were suspended in buffer A* (2% acetonitrile [v/v], 0.1% trifluoroacetic acid [v/v]) and sonicated for 30 min (Branson Ultrasonics, Ultrasonic Cleaner Model 2510).

### Metaproteomics

Samples were analysed using a liquid chromatography (LC) system coupled to a mass spectrometer (MS). The LC was an EASY‐nLC 1200 ultra‐high pressure system (Thermo Fisher Scientific) and was coupled to a Q Exactive HFX Orbitrap MS (Thermo Fisher Scientific) using a nano‐electrospray ion source (Thermo Fisher Scientific). Purified peptides were separated on 50 cm HPLC‐columns (ID: 75 μm; in‐house packed into the tip with ReproSil‐Pur C18‐AQ 1.9 μm resin [Dr. Maisch GmbH]). For each LC–MS/MS analysis about 500 ng peptides were separated on 100 min gradients.

Peptides were separated with a two‐buffer‐system consisting of buffer A (0.1% [v/v] formic acid) and buffer B (0.1% [v/v] formic acid, 80% [v/v] acetonitrile). Peptides were eluted with a linear 70 min gradient of 2–24% buffer B, followed stepwise by a 21 min increase to 40% buffer B, a 4 min increase to 98% buffer B and a 5 min wash of 98% buffer B. The flow rate was constant at 350 nl/min. The temperature of the column was kept at 60°C by an in‐house‐developed oven containing a Peltier element, and parameters were monitored in real time by the SprayQC software (Scheltema & Mann, 2012).

First, data‐dependent acquisition (DDA) was performed of each single organism to establish a library for the data independent acquisition (DIA) of the community culture samples. The DDA scans consisted of a Top15 MS/MS scan method. Target values for the full scan MS spectra were 3e6 charges in the 300–1,650 *m*/*z* range with a maximum injection time of 25 ms and a resolution of 60,000 at *m*/*z* 200. Fragmentation of precursor ions was performed by higher‐energy C‐trap dissociation (HCD) with a normalised collision energy of 27 eV. MS/MS scans were performed at a resolution of 15,000 at *m*/*z* 200 with an ion target value of 5e4 and a maximum injection time of 120 ms. Dynamic exclusion was set to 30 s to avoid repeated sequencing of identical peptides.

MS data for the community culture samples were acquired with the DIA scan mode. Full MS scans were acquired in the range of *m*/*z* 300–1,650 at a resolution of 60,000 at *m*/*z* 200 and the automatic gain control (AGC) set to 3e6. The full MS scan was followed by 32 MS/MS windows per cycle in the range of *m*/*z* 300–1,650 at a resolution of 30,000 at *m*/*z* 200. A higher‐energy collisional dissociation MS/MS scans was acquired with a stepped normalised collision energy of 25/27.5/30 eV and ions were accumulated to reach an AGC target value of 3e6 or for a maximum of 54 ms.

The MS data of the single organisms and of the community cultures were used to generate a DDA‐library and the direct‐DIA‐library, respectively, which were computationally merged into a hybrid library using the Spectronaut software (Biognosys AG). All searches were performed against a merged protein FASTA file of the reference genomes annotated using Prokka (see above). Searches used carbamidomethylation as fixed modification and acetylation of the protein N‐terminus and oxidation of methionines as variable modifications. Trypsin/P proteolytic cleavage rule was used, permitting a maximum of 2 missed cleavages and a minimum peptide length of 7 amino acids. The Q‐value cut‐offs for both library generation and DIA analyses were set to 0.01.

### Metabolomics measurements

Untargeted metabolomics analysis of cell‐free supernatants by flow injection‐mass spectrometry was performed as described previously (Fuhrer *et al*, 2011). Briefly, samples were analysed on a LC/MS platform consisting of a Thermo Scientific Ultimate 3000 LC system with autosampler temperature set to 10°C coupled to a Thermo Scientific Q‐Exactive Plus Fourier transform MS equipped with a heated electrospray ion source and operated in negative or positive ionisation mode. The isocratic flow rate was 150 μl/min of mobile phase consisting of 60:40% (v/v) isopropanol:water buffered with 1 mM ammonium fluoride at pH 9 for negative ionisation mode or 60:40% (v/v) methanol:water buffered with 0.1% formic acid at pH 2 for positive ionisation mode, in both cases containing 10 nM taurocholic acid and 20 nM homotaurine as lock masses. Of note, the LC system was only used to transfer samples from the autosampler to the MS, but did not include a chromatographic column for analyte separation (‘flow injection’). Mass spectra were recorded in profile mode from 50 to 1,000 *m*/*z* with the following instrument settings: sheath gas, 35 a.u.; aux gas, 10 a.u.; aux gas heater, 200°C; sweep gas, 1 a.u.; spray voltage, −3 kV (negative mode) or 4 kV (positive mode); capillary temperature, 250°C; S‐lens RF level, 50 a.u; resolution, 70 k @ 200 m/z; AGC target, 3 × 10^6^ ions, max. inject time, 120 ms; acquisition duration, 60 s. Spectral data processing including peak detection and alignment was performed using an automated pipeline in R analogous to previously published pipelines (Fuhrer *et al*, 2011). To evaluate the impact of the drugs on measurements of other metabolites, we also prepared non‐bacterial controls (i.e., each drug incubated in mGAM culture medium) and analysed them with the same procedure. Detected ions were tentatively annotated as metabolites based on accurate mass within a dynamic tolerance depending on local instrument resolving power ranging from 1 mDa at *m*/*z* = 50 to 5 mDa at *m*/*z* = 1,000 using the Human Metabolome Database (Wishart *et al*, 2018) as reference considering [M‐H] and [M‐2H] ions in negative mode or [M+], [M + H], [M + Na] and [M + K] ions in positive mode and up to two ^12^C to ^13^C substitutions. We additionally provide mappings to the Microbial Metabolites Database (Wishart *et al*, 2023) as part of the associated data repository. Of note, this approach precludes the resolution of isomers, of metabolites mapping to the same ion using different adduct assumptions, of unaccounted neutral gains or losses, or of metabolites with slightly distinct masses that nevertheless map to the same ion within the respective local matching tolerance.

### Metabolomics data analysis

Raw intensity values were quantile‐normalised separately for ions acquired in positive and negative modes. For further analysis, the data from the two acquisition polarity modes were combined in one table and filtered as follows: only annotated ions were retained; ions annotated to ^13^C‐compounds only were removed; for each metabolite, only the ion with the annotation considered most likely was retained (either the ion with the highest correlation with the total ion current, or the ion with the largest mean intensity across samples). We provide both the filtered and the original unfiltered table with metabolite annotations in the associated data repository.

### Gene, transcript and protein counting

Metagenomic and metatranscriptomic reads were mapped against a database of reference genomes containing only the species used in this study, using NGLess and samtools, with a minimum match size of 45 and minimum identity of 97%. Abundance estimates were produced by counting the number of reads mapping to each genome included in the study. If a read mapped to multiple genes, the count was distributed to each of the genes (e.g., if a read maps to gene X and gene Y, gene X and gene Y each get a count of 0.5).

Proteins quantification and filtering. Proteins were filtered based on the information from the DDA experiment on which peptides are detected in which single species. Metaproteomics report with protein and peptide quantification obtained from Spectronaut software applied to DIA samples was used as input. For each peptide in the community peptide report file, number of exact protein and species matches was calculated. For each protein, only unique peptides that match to one species were left for quantification. For each protein, the peptides were sorted according to the number of samples in which they were detected. Protein abundance was calculated as the mean of three most commonly measured peptides as suggested before (Ludwig *et al*, 2018). If the number of peptides was < 3, the protein was discarded.

To reduce taxonomic abundance effects in downstream analyses, taxon‐specific scaling was performed on metagenomics, metatranscriptomics and metaproteomics as described by (Klingenberg & Meinicke, 2017). These measurements are all relative, and therefore changes in cell counts or biomass are not taken into account.

### Species abundance estimation

Multiple computational strategies were used to estimate species abundance. Unless stated otherwise, for all analyses the species abundances resulting from read mapping were used. For this approach, first a database of 16S rRNA regions was constructed by manually querying the SILVA rRNA database (Quast *et al*, 2013) and extracting the representative sequence from each of our 32 species. Amplicon sequencing reads were then mapped against this database using MAPseq v1.2.4 (Matias Rodrigues *et al*, 2017). Paired reads were mapped independently and assignments were only considered upon agreement. Abundance estimates were then produced by counting the number of reads mapping to each genome included in the study. For metagenome derived estimates, total counts were normalised by the size of the genome (number of base‐pairs). For metatranscriptome derived estimates, additional steps were required. Gene predictions by Prokka/Prodigal were used to calculate the total number of coding bases per genome, after exclusion of rRNA regions. Finally, total read counts were normalised by the number of coding bases on each genome.

Species abundance was estimated from metaproteomic data by summing up all filtered protein intensities detected per each species, and dividing the sum by the total summed protein intensity in a given sample.

In addition, to the approaches based on read mapping, several popular tools were used to estimate species abundance. For amplicon sequencing, DADA2 v1.10 (Callahan *et al*, 2016) was used with the GTDB database release 86 (Parks *et al*, 2018) for sequence classification which was limited to genus level classification. Metagenomic and metatranscriptomic species abundances were estimated using mOTUs v2.5 (Milanese *et al*, 2019) and MetaPhlAn v3 (Beghini *et al*, 2021).

### Coverage analyses

Gene, transcript and protein coverage were defined as the number of genes/transcripts/proteins that showed a count higher than 0, divided by the total number of predicted genes per species. For pathway coverage, the same approach was used, but genes/transcripts/proteins were grouped by the KEGG pathways instead and thus divided by the number of KEGG orthologs in one single pathway. The same procedure was repeated for metabolites, but using the number of metabolites per pathway as predicted by KEGG instead of the number of KEGG orthologs.

### Mantel test

Mantel tests were performed to compare each pair of omics datasets and evaluate the similarity between them. Abundance tables of each omics were transformed into distance matrices using 1−Spearman's correlation coefficient, and the matrices were compared using the mantel function in the vegan package (version 2.5.5) with the default option. For gene (metagenomic), transcript (metatranscriptomic) and protein (metaproteomic) level profiles, features with mean abundances below 1E‐7, 1E‐7 and 1E‐5, respectively, were excluded, and only features above those thresholds were included in the analysis. All the features were included in the species‐level profiles for each omics. Sixty‐one samples that were common among all the omics datasets were used in this analysis.

### Differential species abundance analysis

Differential analysis of species abundance across conditions was performed with ANCOM v. 2.1. Tables of species abundances calculated from each omics measurements were preprocessed with feature_table_pre_process with sample names used as sample variables, condition used as group variable, and parameters out_cut = 0.05; zero_cut = 0.90; lib_cut = 0; neg_lb = TRUE. The ANCOM function was applied to each pre‐processed table with condition used as the main variable and time used as the formula for adjustment (with parameters: main_var = “condition’; p_adj_method = ‘BH’; alpha = 0.05, adj_formula = “time”; rand_formula = NULL). *P*‐values were adjusted with Benjamini–Hochberg method (p_adj_method = ‘BH’). The cut‐off of 0.7 for the W statistic was used to identify significantly differentially abundant species (detected_0.7 = TRUE).

### Differential transcript, protein and metabolite abundance analysis

Differential transcript analysis was performed using DESeq2 v1.26.0 (Love *et al*, 2014) after taxon‐specific scaling (see above). The design formula included the factors run, drug, time point and the interaction term drug:timepoint. Statistical testing was performed with the Wald‐test and IHW (Ignatiadis *et al*, 2016) to control the false discovery rate.

Differential protein and metabolite analysis were performed using repeated measures analysis of variance using the lmer function in the ade4 package. The same formula used in the differential transcript analysis was also used in the analysis. To exclude low‐abundant features, those that have 0 or NA in at least half of the samples were removed prior to the analysis. *P*‐values were adjusted by the IHW method. Fold changes of proteins and metabolites compared to those of controls were calculated based on raw values.

### Pathway and COG enrichment analysis

Pathway enrichment was performed on differentially abundant features (cut‐off for metatranscriptomics abs(log_2_(fold change)) > 2, pFDR < 0.001, cut‐off for metabolomics and metaproteomics abs(log_2_(fold change)) > log_2_(1.5), pFDR < 0.05) with Fisher exact test using stats.fisher_exact in Python 3.7.7. *P*‐values were adjusted with Benjamini–Hochberg procedure with multipletests function from statsmodels. For metabolomics, pathway enrichment analysis was performed for ion features rather than metabolite features (e.g. if one ion is annotated to two or more metabolites from the same pathway, it is counted only once in pathway enrichment analysis). For each feature, only one measurement corresponding to the maximum absolute fold change over time was used for pathway enrichment analysis. COG enrichment was performed in the R environment using ClusterProfiler (Wu *et al*, 2021).

## Author contributions

**Sander Wuyts:** Data curation; software; formal analysis; visualization; methodology; writing – original draft; writing – review and editing. **Renato Alves:** Conceptualization; data curation; software; formal analysis; visualization; methodology; writing – original draft; writing – review and editing. **Maria Zimmermann‐Kogadeeva:** Data curation; software; formal analysis; visualization; methodology; writing – original draft; writing – review and editing. **Suguru Nishijima:** Data curation; software; formal analysis; visualization; methodology; writing – original draft; writing – review and editing. **Sonja Blasche:** Investigation; writing – review and editing. **Marja Driessen:** Conceptualization; investigation. **Philipp E Geyer:** Investigation; writing – review and editing. **Rajna Hercog:** Investigation. **Ece Kartal:** Investigation; writing – review and editing. **Lisa Maier:** Conceptualization. **Johannes B Müller:** Investigation. **Sarela Garcia Santamarina:** Investigation; writing – review and editing. **Thomas Sebastian B Schmidt:** Software; formal analysis; writing – review and editing. **Daniel C Sevin:** Formal analysis; investigation; writing – review and editing. **Anja Telzerow:** Investigation. **Peter V Treit:** Investigation; writing – review and editing. **Tobias Wenzel:** Investigation. **Athanasios Typas:** Conceptualization; supervision; writing – original draft; writing – review and editing. **Kiran R Patil:** Conceptualization; supervision; writing – original draft; writing – review and editing. **Matthias Mann:** Supervision. **Michael Kuhn:** Conceptualization; formal analysis; supervision; visualization; writing – original draft; writing – review and editing. **Peer Bork:** Conceptualization; supervision; funding acquisition; writing – original draft; writing – review and editing.

## Disclosure and competing interests statement

The authors declare no competing interests. PB, AT and MM are members of the Editorial Advisory Board of Molecular Systems Biology. This has no bearing on the editorial consideration of this article for publication.

## Supporting information



Appendix S1

Expanded View Figures PDF

PDF+

## Data Availability

The MS‐based proteomics data have been deposited to the ProteomeXchange Consortium via the PRIDE partner repository and are available via ProteomeXchange with identifier PXD036445. Metabolomic data has been submitted to MetaboLights under accession number MTBLS3129. Sequencing data is deposited at the European Nucleotide Archive (ENA): PRJEB46619. Preproccessed data files and tables are available on Figshare at https://doi.org/10.6084/m9.figshare.21667763. Code to generate all figures is available at https://github.com/grp‐bork/multiomics_Wuyts_2022.
