# Supplementary material for: Consistency across multi‐omics layers in a drug‐perturbed gut microbial community
Source: Mol Syst Biol. 2023 Jul 24;19(9):e11525. doi: 10.15252/msb.202311525 (PMC10495815; doi:10.15252/msb.202311525)
Supplement: Supplementary file 1 — Appendix S1 [file MSB-19-e11525-s002.pdf]

Appendix

Appendix Table 1: Species used in this study 2

Appendix Figure 1: High technical reproducibility across all omics methods 3

Appendix Figure 2: Comparison of species abundance estimation by different pipelines ... 4

Appendix Figure 3: Comparison of gene abundance estimates between different omics ... 5

Appendix Figure 4: Community composition in different conditions becomes more similar at later time points 6

Appendix Figure 5: Metabolites increased upon metformin exposure are likely to be measurement artifacts 7

Appendix Figure 6: COG categories of genes regulated during early exposure to drugs 8

Appendix Figure 7: Correlation between transcript and protein fold changes across time points 9

Appendix Figure 8: Chlorpromazine causes upregulation of heat shock related genes in *Escherichia coli* 10

Appendix Table 1: Species used in this study

Identifier	Species	GTDB 95 species name	Strain	Source	Gram stain
NT5021	Akkermansia muciniphila	Akkermansia muciniphila	type strain, Muc	DSM No.: 22959	negative
NT5003	Bacteroides fragilis	Bacteroides fragilis	EN-2, VPI 2553	DSM No.: 2151	negative
NT5004	Bacteroides thetaiotaomicron	Bacteroides thetaiotaomicron	E50(VPI 5482)	DSM No.: 2079	negative
NT5002	Bacteroides uniformis	Bacteroides uniformis	VPI 0061	DSM No.: 6597	negative
NT5022	Bifidobacterium adolescentis	Bifidobacterium adolescentis	type strain, E194a (Variant a)	DSM No.: 20083	positive
NT5028	Bifidobacterium longum	Bifidobacterium longum	type strain, E194b (Variant a)	DSM No.: 20219	positive
NT5036	Bilophila wadsworthia	Bilophila wadsworthia	type strain, WAL 7959 [Lab 88-130H]	ATCC No.: 49260	negative
NT5069	Blautia obeum	Blautia_A obeum	type strain	DSM No.: 25238	positive
NT5026	Clostridium bolteae	Clostridium_M bolteae	type strain, WAL 16351	DSM No.: 15670	positive
NT5032	Clostridium perfringens	Clostridium_P perfringens	C36	DSM No.: 11782	positive
NT5006	Clostridium ramosum	Erysipelatoclostridium ramosum	type strain, 113-I, VPI 0427	DSM No.: 1402	positive
NT5037	Clostridium saccharolyticum	Hungatella saccharolyticum	type strain, WM1	DSM No.: 2544	positive
NT5073	Collinsella aerofaciens	Collinsella aerofaciens	type strain, ATCC 25986, VPI 1003	DSM No.: 3979	positive
NT5048	Coprococcus comes	Coprococcus_B comes	type strain, VPI CI-38	ATCC No.: 27758	positive
NT5076	Dorea formicigenerans	Dorea formicigenerans	VPI C8-13	DSM No.: 3992	positive
NT5024	Eggerthella lenta	Eggerthella lenta	type strain, 1899 B, VPI 0255	DSM No.: 2243	positive
NT5078	Escherichia coli	Escherichia coli	ED1a	Denamur Lab (INSERM)	negative
NT5009	Eubacterium rectale	Agathobacter rectale	A1-86	DSM No.: 17629	positive
NT5025	Fusobacterium nucleatum	Fusobacterium nucleatum	type strain, 1612A, VPI 4355	DSM No.: 15643	negative
NT5042	Lactobacillus paracasei	Lactobacillus_C paracasei	LPC-37, ATCC No.: SD5275	Dupont Health and Nutrition	positive
NT5081	Odoribacter splanchnicus	Odoribacter splanchnicus	type strain, 1651/6	DSM 20712	negative
NT5074	Parabacteroides distasonis	Parabacteroides distasonis	ATCC 8503, CCUG 4941, JCM 5825, NCTC 11152	DSM No.: 20701	negative
NT5071	Parabacteroides merdae	Parabacteroides merdae	VPI T4-1, CIP 104202T, JCM 9497	DSM No.: 19495	negative
NT5001	Phocaeicola vulgatus	Bacteroides_B vulgatus	type strain	DSM No.: 1447	negative
NT5019	Prevotella copri	Prevotella copri	type strain, CB7	DSM No.: 18205	negative
NT5011	Roseburia intestinalis	Roseburia intestinalis	L1-82	DSM No.: 14610	positive
NT5045	Ruminococcus bromii	Ruminococcus_E bromii	type strain, VPI 6883	ATCC No.: 27255	positive
NT5046	Ruminococcus gnavus	Faecalicatena gnavus	type strain, VPI C7-9	ATCC No.: 29149	positive
NT5047	Ruminococcus torques	Faecalicatena torques	type strain, VPI B2-51	ATCC No.: 27756	positive
NT5072	Streptococcus parasanguinis	Streptococcus parasanguinis	type strain	DSM No.: 6778	positive
NT5038	Streptococcus salivarius	Streptococcus salivarius	type strain, 275	DSM No.: 20560	positive
NT5017	Veillonella parvula	Veillonella parvula	type strain, Te3	DSM No.: 2008	negative

Appendix Figure 1: High technical reproducibility across all omics methods

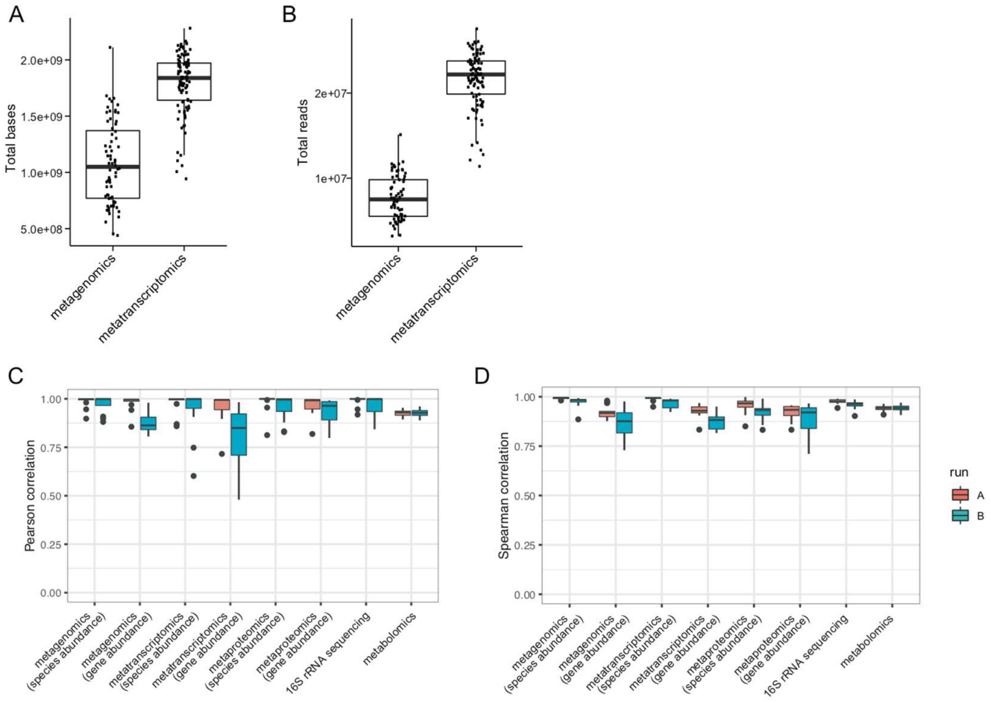

A. Total bases detected across metagenomics and metatranscriptomic samples.
B. Total reads detected across metagenomics and metatranscriptomic samples.
C. Pearson correlation coefficients between technical replicates in runs A and B for each omics measurement.
D. Spearman correlation coefficients between technical replicates in runs A and B for each omics measurement.

Appendix Figure 2: Comparison of species abundance estimation by different pipelines

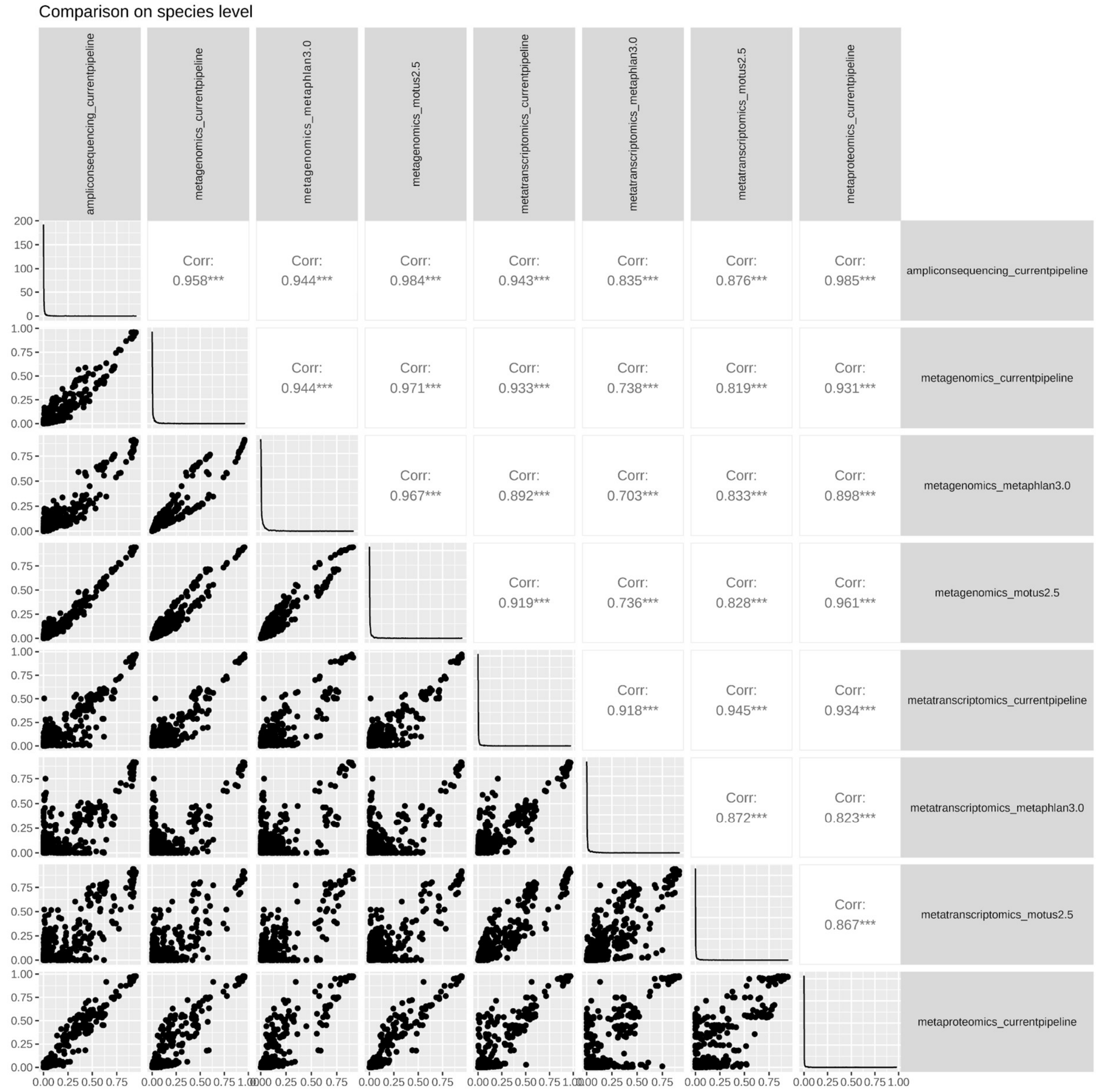

Each dot depicts abundance of one species in one of the four conditions and time points estimated by different methods.

Appendix Figure 3: Comparison of gene abundance estimates between different omics

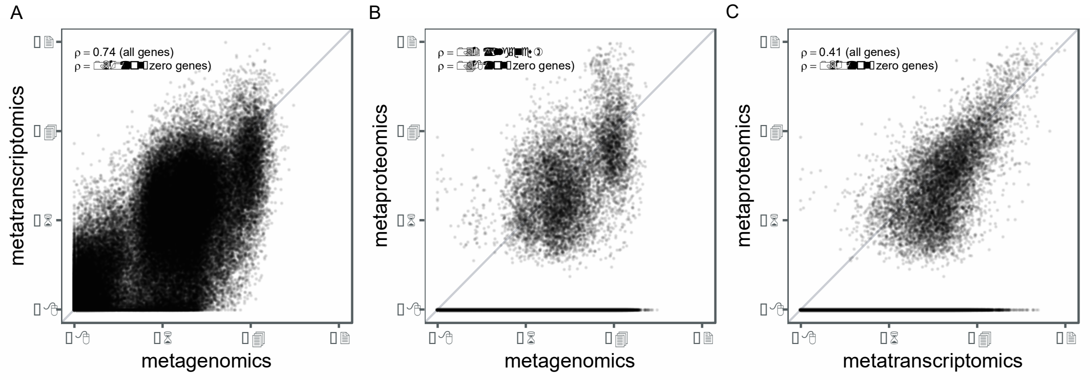

Each point corresponds to one gene estimate in one of the four conditions, as detected by A. metagenomics vs metatranscriptomics, B. metagenomics vs metaproteomics, or C. metatranscriptomics vs metaproteomics measurements.

Appendix Figure 4: Community composition in different conditions becomes more similar at later time points

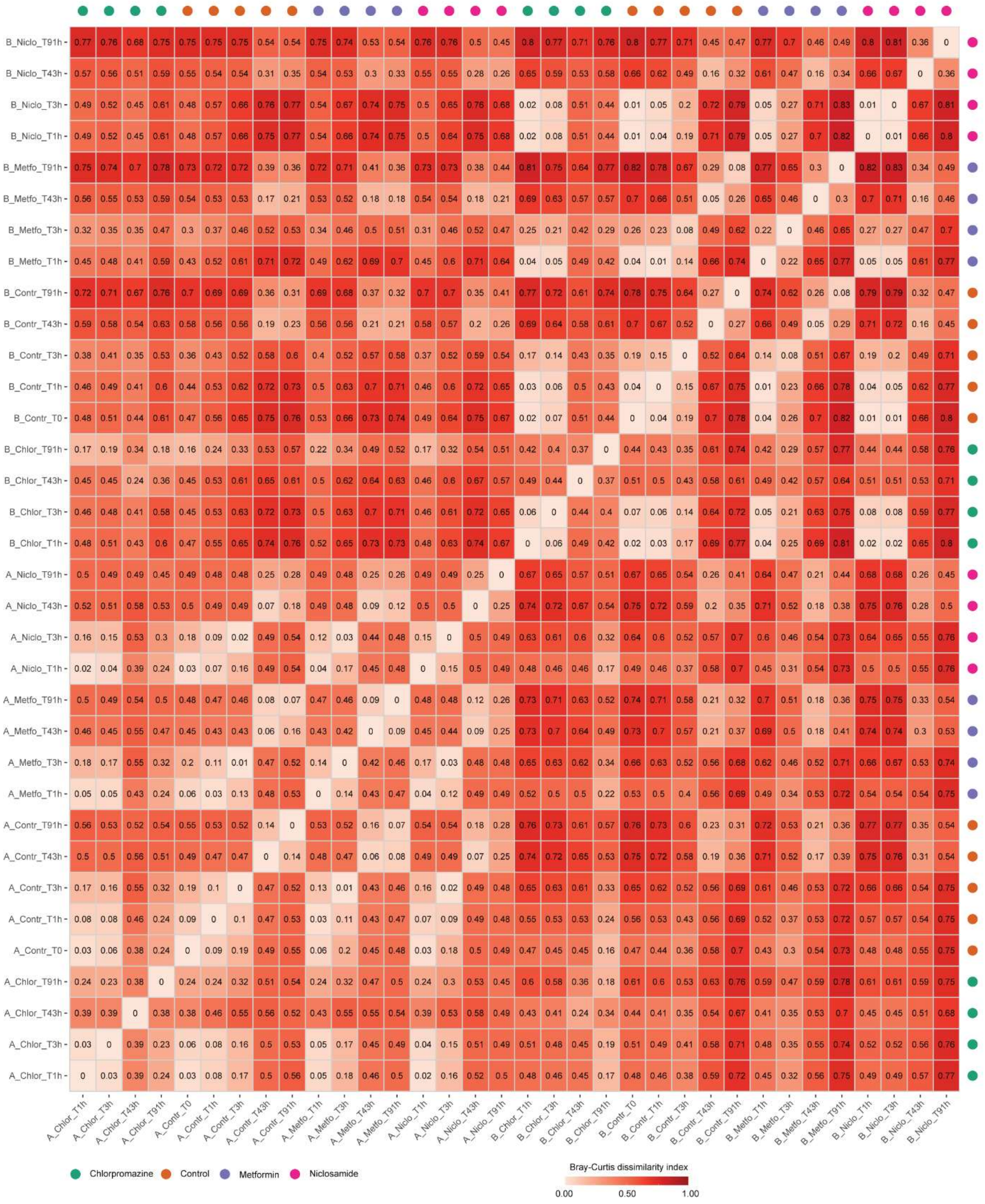

Bray-Curtis dissimilarity values for pairwise comparison of community compositions at each time point after each of the drug treatments in runs A and B.

Appendix Figure 5: Metabolites increased upon metformin exposure are likely to be measurement artifacts

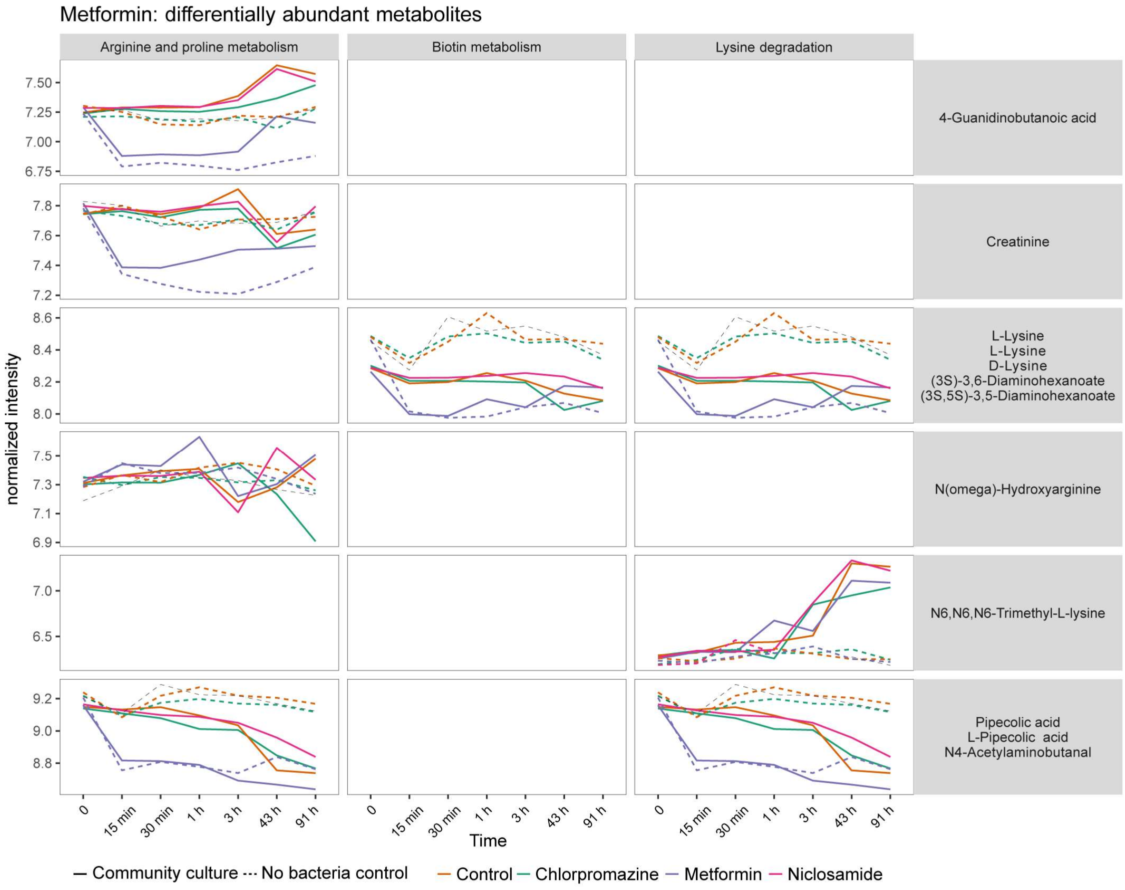

Metabolite profiles overtime after drug treatment in community samples and non-bacteria controls. Metabolites depicted were selected from pathways significantly differentially abundant on metformin revealed by pathway enrichment analysis.

Appendix Figure 6: COG categories of genes regulated during early exposure to drugs

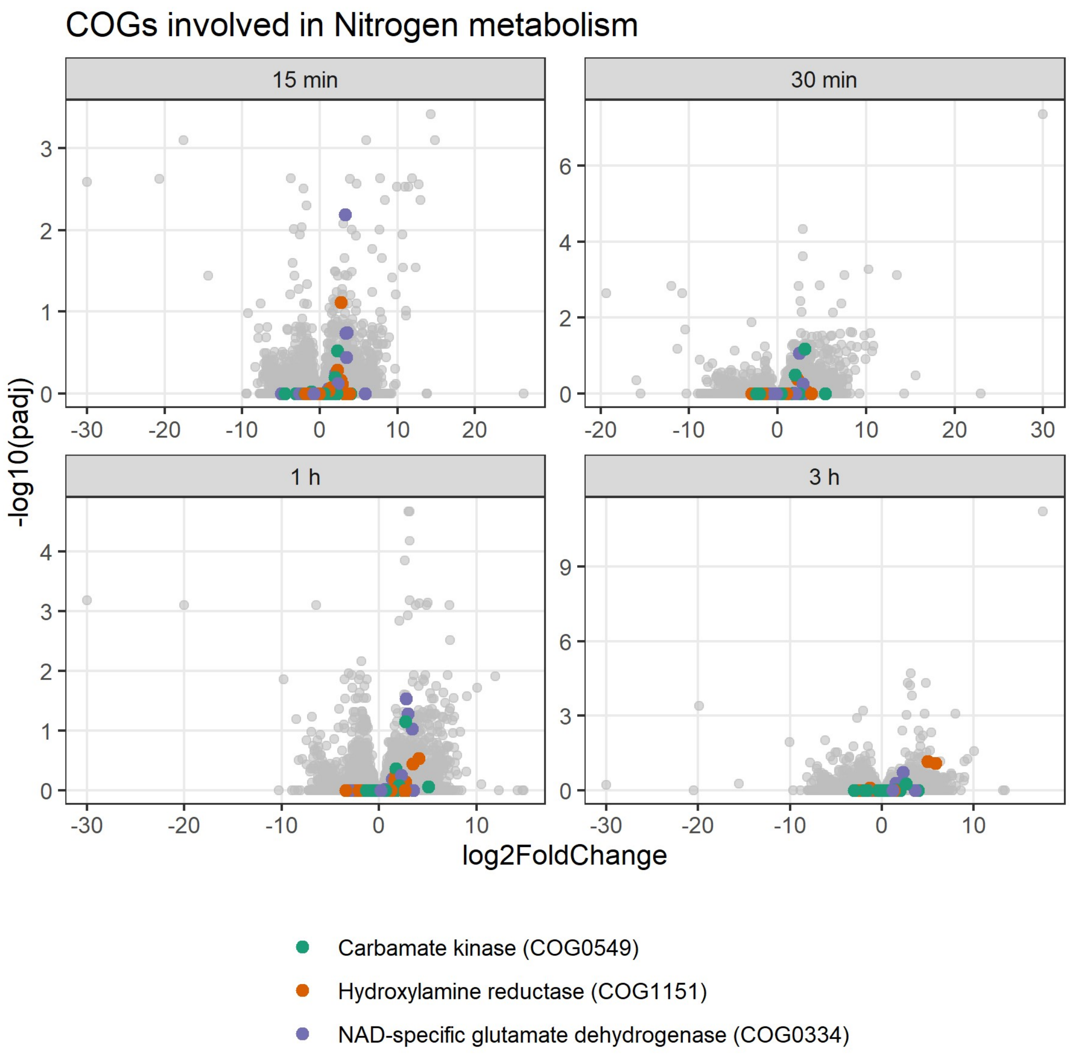

Volcano plots depicting fold change (log2 scale) and significance (-log10 FDR) of COG abundance upon niclosamide treatment in the first four time points.

Appendix Figure 7: Correlation between transcript and protein fold changes across time points

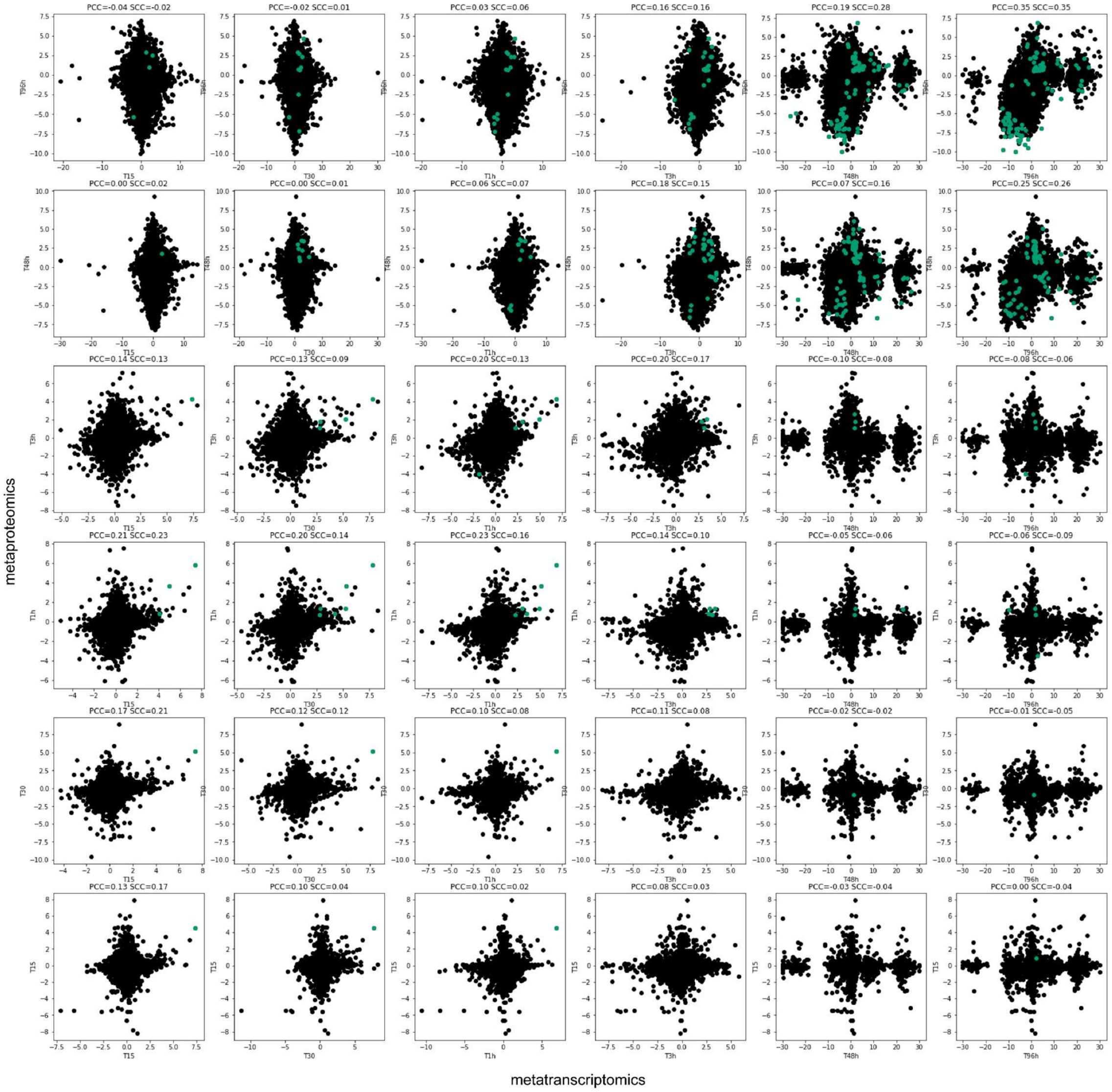

Only proteins detected both by metatranscriptomics and metaproteomics are depicted. Proteins passing the significance FDR cutoff of 0.1 both in proteomics and transcriptomics measurements are depicted in green. PCC - Pearson correlation coefficient, SCC - Spearman correlation coefficient.

Appendix Figure 8: Chlorpromazine causes upregulation of heat shock related genes in *Escherichia coli*

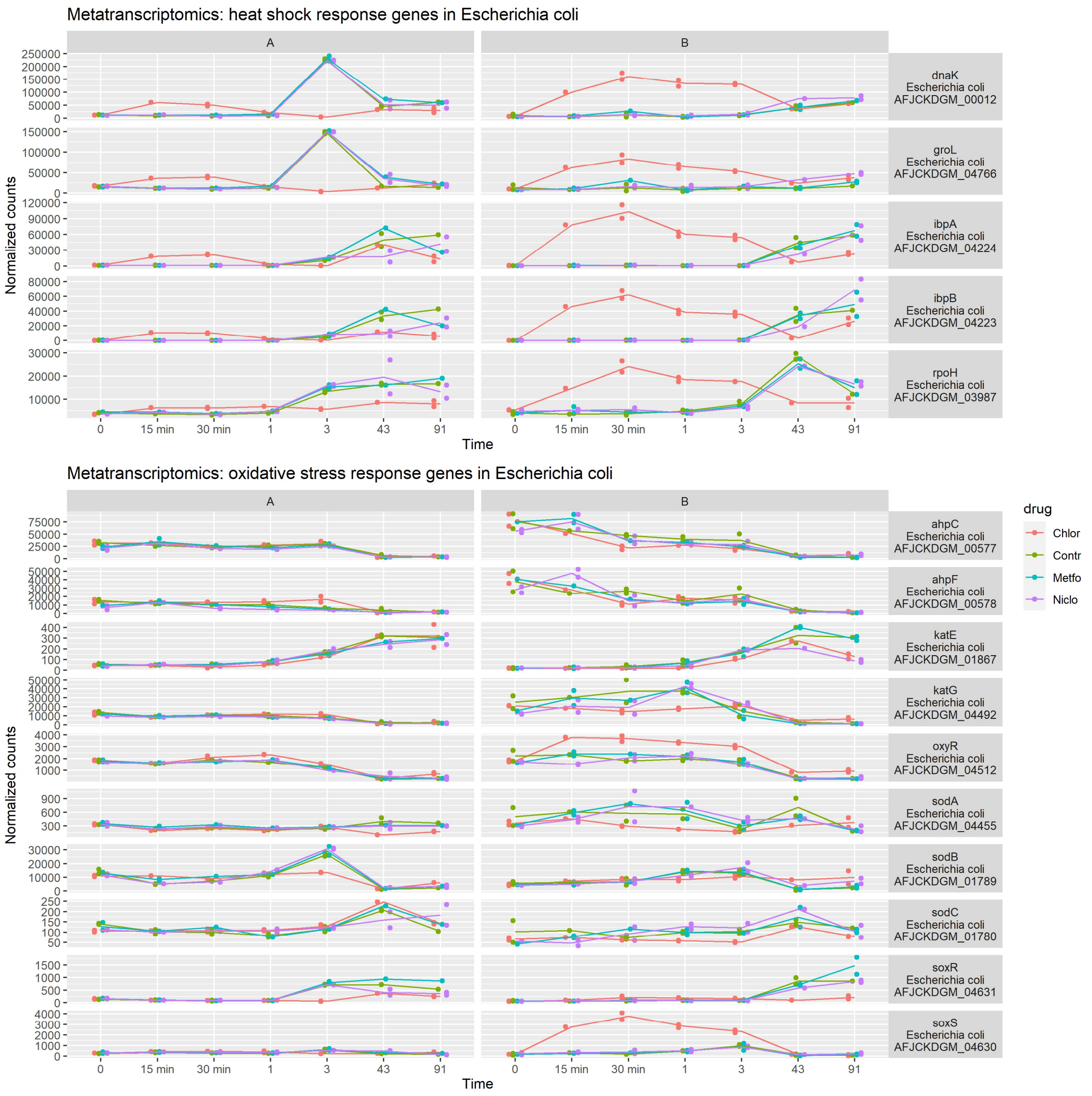

Only a small number genes related to oxidative stress are upregulated in chlorpromazine treatment ("Chlor" in the legend).
