# Supplementary material for: Consistency across multi‐omics layers in a drug‐perturbed gut microbial community
Source: Mol Syst Biol. 2023 Jul 24;19(9):e11525. doi: 10.15252/msb.202311525 (PMC10495815; doi:10.15252/msb.202311525)
Supplement: Supplementary file 2 — Expanded View Figures PDF [file MSB-19-e11525-s001.pdf]

Expanded View Figures

Figure EV1. Establishment of a stable microbial community after three community transfers.

A Relative abundance of the community members during the community transfer phase prior to drug treatment.
B Alpha-diversity measurements during the community transfer phase prior to drug treatment.
C The Bray–Curtis dissimilarity values for pairwise comparison of community compositions during the community transfer phase prior to drug treatment.
D Growth curves were measured every hour during community establishment. We fit a sigmoid function to the measurements per day, and normalised the resulting OD curves. Based on the observed growth curve, we chose to treat the community after 5 h (black vertical line) so that the tightly spaced time points within 3 h are all within the exponential phase.

 © 2023 The Authors

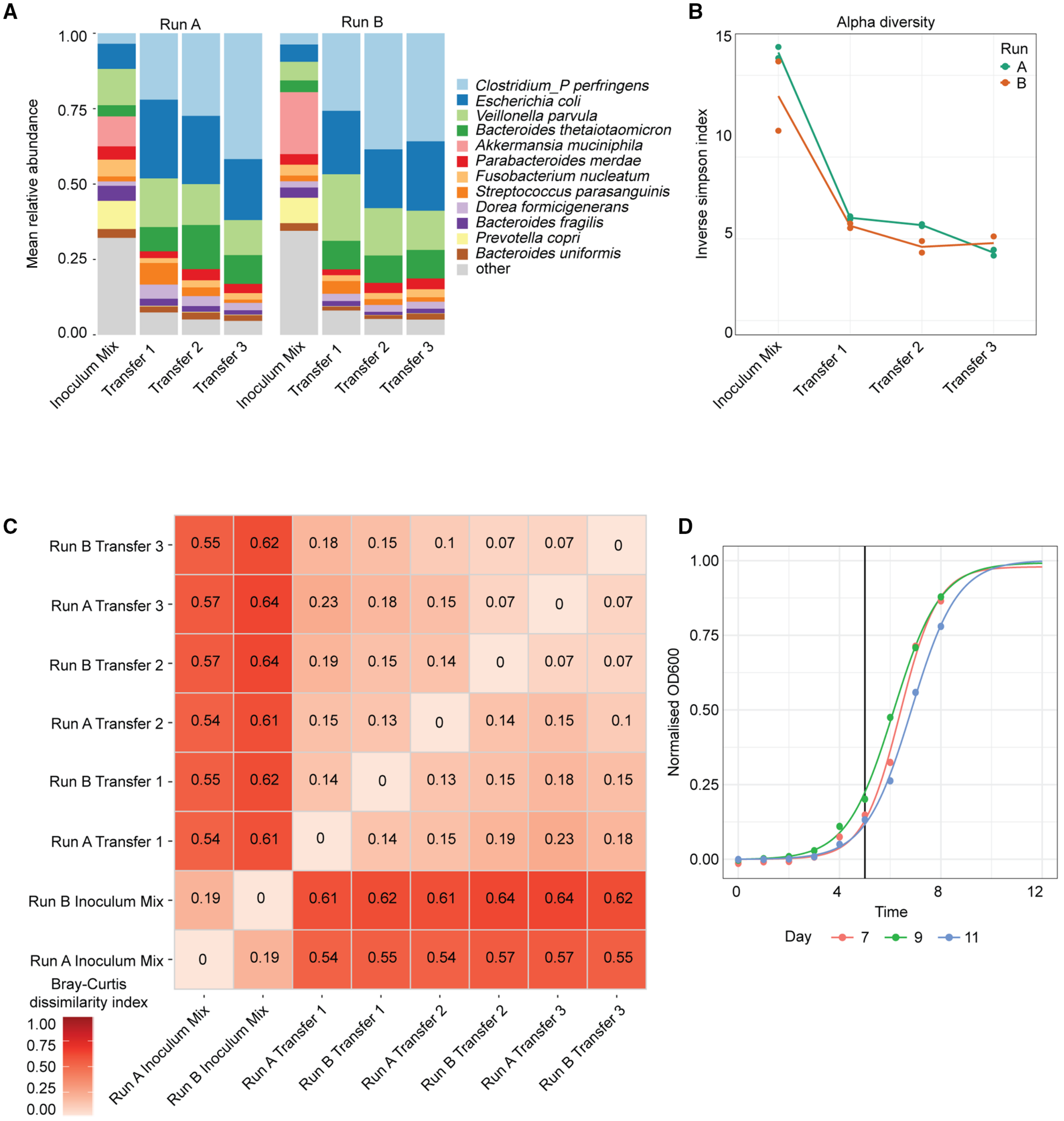

Figure EV1.

Figure EV2. Differences between species abundances estimated by metagenomics and 16S sequencing are not species-, condition- or Gram-type specific.

A Metagenomics versus 16S sequencing species abundances coloured by species.

B Metagenomics versus 16S sequencing species abundances coloured by condition.

C Metagenomics versus 16S sequencing species abundances coloured by Gram staining.

© 2023 The Authors

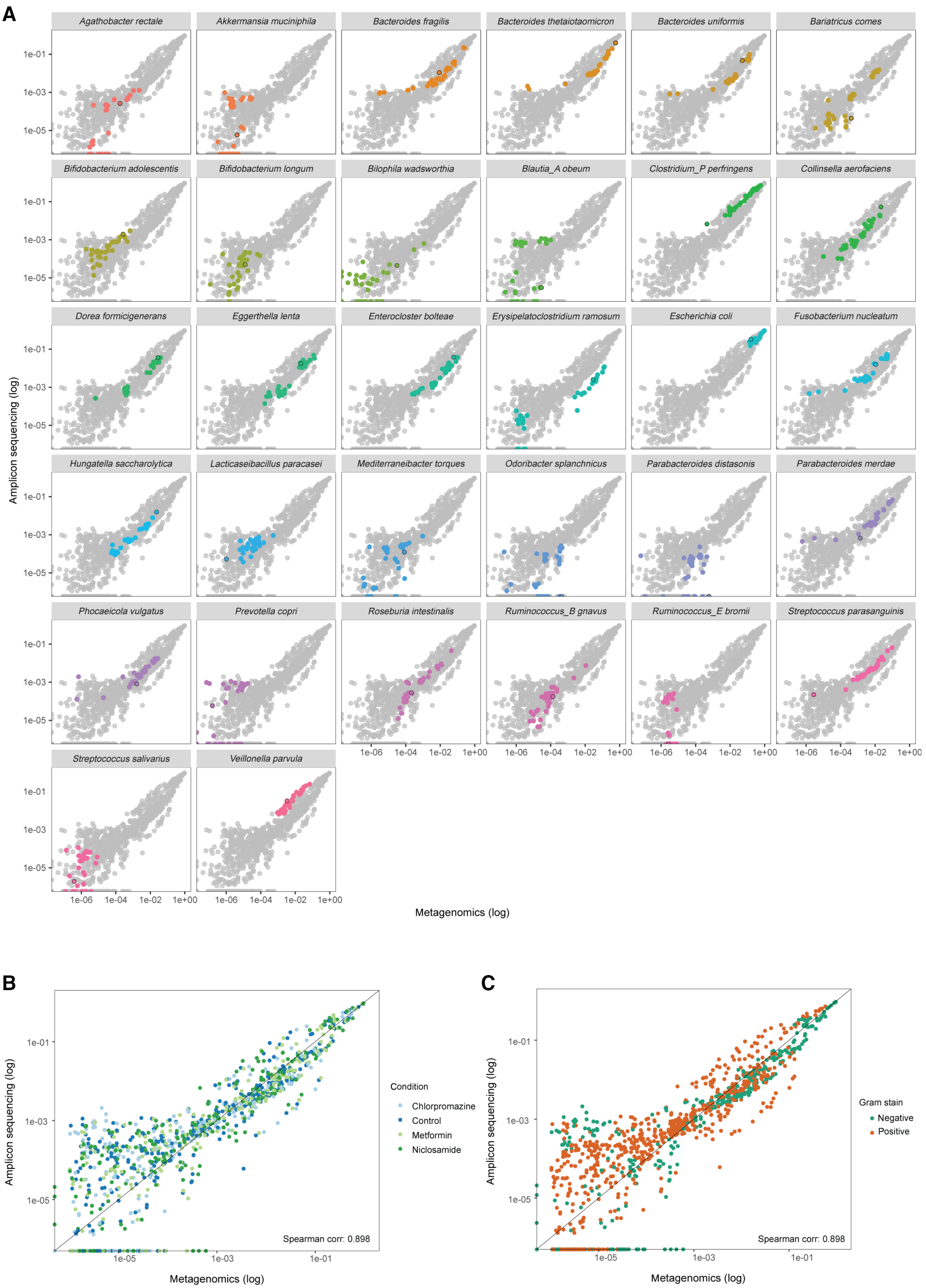

Figure EV2.

© 2023 The Authors

Figure EV3. Chlorpromazine strongly affects community composition.

A, B Community alpha diversity measurements over time after drug treatment for runs A and B, correspondingly.

C Relative species abundance changes over time in the three drug conditions and control. Relative abundance measured from 16S rRNA amplicon sequencing, metatranscriptomic and metaproteomic data.

© 2023 The Authors

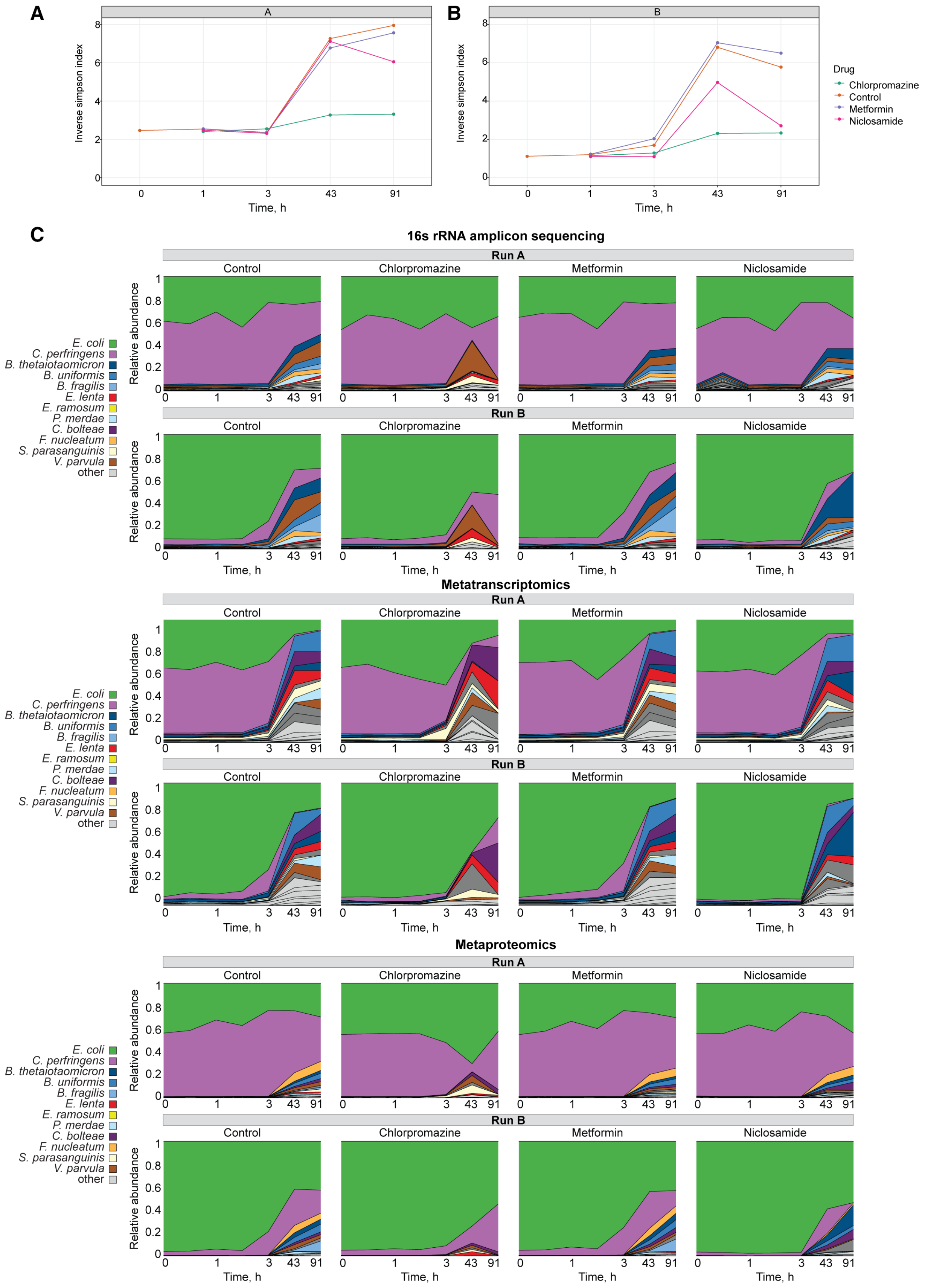

Figure EV3.

 © 2023 The Authors

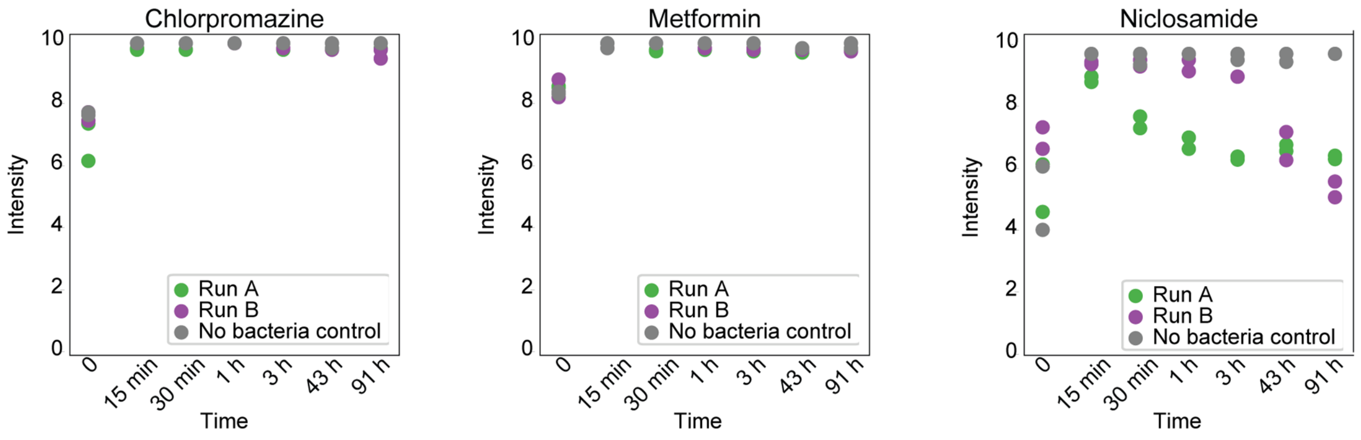

Figure EV4. Drug profiles measured over time.

Drug concentrations were measured both during the community experiments and in controls in sterile medium.

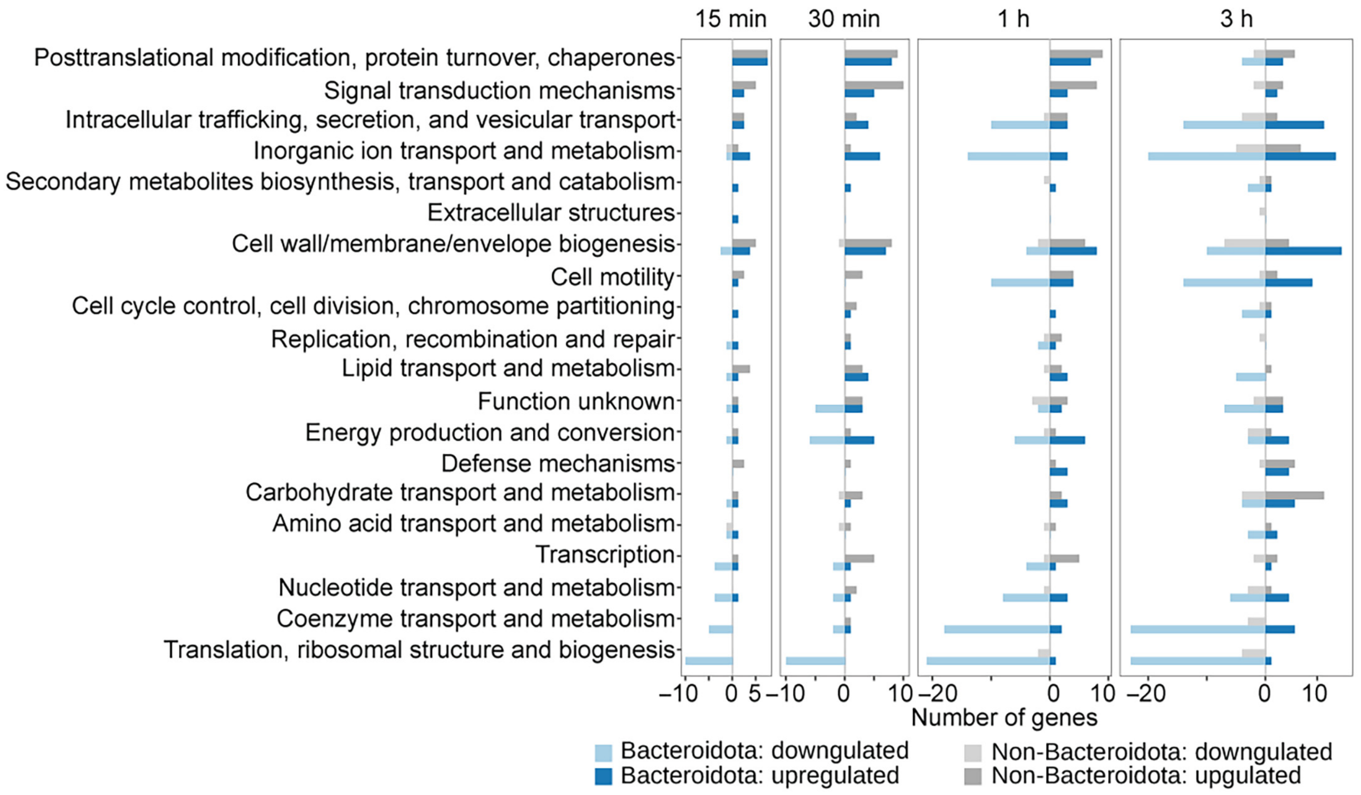

Figure EV5. Number of protein-coding genes grouped by COG category changing per time point upon chlorpromazine treatment.

Bacteroidota species quickly downregulated genes involved in translation and the ribosome compared to other species.

© 2023 The Authors
