# Supplementary material for: Consistency across multi‐omics layers in a drug‐perturbed gut microbial community
Source: Mol Syst Biol. 2023 Jul 24;19(9):e11525. doi: 10.15252/msb.202311525 (PMC10495815; doi:10.15252/msb.202311525)
Supplement: Supplementary file 4 [file MSB-19-e11525-s004.pdf]

molecular systems biology

Consistency across multi-omics layers in a drug-perturbed gut microbial community

Sander Wuyts, Renato Alves, Maria Zimmermann-Kogadeeva, Suguru Nishijima, Sonja Blasche, Marja Driessen, Philipp Geyer, Rajna Hercog, Ece Kartal, Lisa Maier, Johannes Müller, Sarela Garcia Santamarina, Thomas Schmidt, Daniel Sevin, Anja Telzerow, Peter Treit, Tobias Wenzel, Athanasios Typas, Kiran Patil, Matthias Mann, Michael Kuhn, and Peer Bork
DOI: 10.15252/msb.202311525

Corresponding author(s): Peer Bork (peer.bork@embl.org)

Review Timeline:

Submission Date:	9th Jan 23
Editorial Decision:	31st Jan 23
Revision Received:	17th May 23
Editorial Decision:	7th Jun 23
Revision Received:	4th Jul 23
Accepted:	6th Jul 23

Editor: Maria Polychronidou

Transaction Report:

(Note: With the exception of the correction of typographical or spelling errors that could be a source of ambiguity, letters and reports are not edited. Depending on transfer agreements, referee reports obtained elsewhere may or may not be included in this compilation. Referee reports are anonymous unless the Referee chooses to sign their reports.)

31st Jan 2023

Manuscript Number: MSB-2023-11525
Title: Consistency across multi-omics layers in a drug-perturbed gut microbial community

Dear Peer,

Thank you again for submitting your work to Molecular Systems Biology. We have now heard back from the three reviewers who agreed to evaluate your study. As you will see below, the reviewers acknowledge that the addressed topic is interesting. They do however raise a series of concerns, which we would ask you to address in a revision.

I think that the reviewers' recommendations are rather clear and straightforward to address. I therefore see no need to repeat any of the comments listed below. Regarding the issues raised by reviewer #1 on the advance and insights provided by the study, we would ask you to better explain the contribution of the work to understanding drug-perturbed microbial communities and to generally ensure that the advance and new insights provided by the study are clarified.

All issues raised by the referees would need to be satisfactorily addressed. Please let me know in case you would like to discuss in further detail any of the issues raised, I would be happy to schedule a call.

On a more editorial level, we would ask you to address the following points:

- Please provide a .doc version of the manuscript text (including legends for the main figures) and individual production quality figure files for the main Figures (one file per figure).

- We have replaced Supplementary Information by the Expanded View (EV format). In this case, all additional figures and Tables can be included in a PDF called Appendix. Appendix figures and Tables should be labeled and called out as: "Appendix Figure S1, Appendix Figure S2... Appendix Table S1..." etc. Each legend should be below the corresponding Figure/Table in the Appendix. Please include a Table of Contents in the beginning of the Appendix. For detailed instructions regarding expanded view please refer to our Author Guidelines: .

- Please provide a "standfirst text" summarizing the study in one or two sentences (approximately 250 characters), three to four "bullet points" highlighting the main findings and a "synopsis image" (550px width and max 400px height, jpeg format) to highlight the paper on our homepage.

- Please include 5 keywords.

- All Materials and Methods need to be described in the main text. We would encourage you to use 'Structured Methods', our new Materials and Methods format. According to this format, the Material and Methods section should include a Reagents and Tools Table (listing key reagents, experimental models, software and relevant equipment and including their sources and relevant identifiers) followed by a Methods and Protocols section in which we encourage the authors to describe their methods using a step-by-step protocol format with bullet points, to facilitate the adoption of the methodologies across labs. More information on how to adhere to this format as well as downloadable templates (.doc or .xls) for the Reagents and Tools Table can be found in our author guidelines: . An example of a Method paper with Structured Methods can be found here:

- Please include a "Disclosure & Competing Interests Statement".

- Please include a "Data availability" section describing how the data, code etc. have been made available. This section needs to be formatted according to the example below:
The datasets and computer code produced in this study are available in the following databases:
- Chip-Seq data: Gene Expression Omnibus GSE46748 (https://www.ncbi.nlm.nih.gov/geo/query/acc.cgi?acc=GSE46748)
- Modeling computer scripts: GitHub (https://github.com/SysBioChalmers/GECKO/releases/tag/v1.0)
- [data type]: [full name of the resource] [accession number/identifier] ([doi or URL or identifiers.org/DATABASE:ACCESSION])

- For data quantification: please specify the name of the statistical test used to generate error bars and P values, the number (n) of independent experiments (specify technical or biological replicates) underlying each data point and the test used to calculate p-values in each figure legend. The figure legends should contain a basic description of n, P and the test applied. Graphs must include a description of the bars and the error bars (s.d., s.e.m.).

- When you resubmit your manuscript, please download our CHECKLIST (https://bit.ly/EMBOPressAuthorChecklist) and include the completed form in your submission.
Please note that the Author Checklist will be published alongside the paper as part of the transparent process

(https://www.embopress.org/page/journal/17444292/authorguide#transparentprocess).

If you feel you can satisfactorily deal with these points and those listed by the referees, you may wish to submit a revised version of your manuscript. Please attach a covering letter giving details of the way in which you have handled each of the points raised by the referees. A revised manuscript will be once again subject to review and you probably understand that we can give you no guarantee at this stage that the eventual outcome will be favorable.

Kind regards,

Maria

Maria Polychronidou, PhD
Senior Editor
Molecular Systems Biology

We realize that it is difficult to revise to a specific deadline. In the interest of protecting the conceptual advance provided by the work, we recommend a revision within 3 months (1st May 2023). Please discuss the revision progress ahead of this time with the editor if you require more time to complete the revisions. Use the link below to submit your revision:

https://msb.msubmit.net/cgi-bin/main.plex

IMPORTANT: When you send your revision, we will require the following items:
1. the manuscript text in LaTeX, RTF or MS Word format
2. a letter with a detailed description of the changes made in response to the referees. Please specify clearly the exact places in the text (pages and paragraphs) where each change has been made in response to each specific comment given
3. three to four 'bullet points' highlighting the main findings of your study
4. a short 'blurb' text summarizing in two sentences the study (max. 250 characters)
5. a 'thumbnail image' (550px width and max 400px height, Illustrator, PowerPoint or jpeg format), which can be used as 'visual title' for the synopsis section of your paper.
6. Please include an author contributions statement after the Acknowledgements section (see https://www.embopress.org/page/journal/17444292/authorguide)
7. Please complete the CHECKLIST available at (https://bit.ly/EMBOPressAuthorChecklist).
Please note that the Author Checklist will be published alongside the paper as part of the transparent process (https://www.embopress.org/page/journal/17444292/authorguide#transparentprocess).
8. When assembling figures, please refer to our figure preparation guideline in order to ensure proper formatting and readability in print as well as on screen:
https://bit.ly/EMBOPressFigurePreparationGuideline
See also figure legend guidelines: https://www.embopress.org/page/journal/17444292/authorguide#figureformat
9. Please note that corresponding authors are required to supply an ORCID ID for their name upon submission of a revised manuscript (EMBO Press signed a joint statement to encourage ORCID adoption).
(https://www.embopress.org/page/journal/17444292/authorguide#editorialprocess)
Currently, our records indicate that the ORCID for your account is 0000-0002-2627-833X.

Please click the link below to modify this ORCID:
Link Not Available

10. At EMBO Press we ask authors to provide source data for the main manuscript figures. Our source data coordinator will contact you to discuss which figure panels we would need source data for and will also provide you with helpful tips on how to upload and organize the files.

The system will prompt you to fill in your funding and payment information. This will allow Wiley to send you a quote for the article processing charge (APC) in case of acceptance. This quote takes into account any reduction or fee waivers that you may be eligible for. Authors do not need to pay any fees before their manuscript is accepted and transferred to the publisher.

EMBO Press participates in many Publish and Read agreements that allow authors to publish Open Access with reduced/no publication charges. Check your eligibility: https://authorservices.wiley.com/author-resources/Journal-Authors/open-access/affiliation-policies-payments/index.html

As a matter of course, please make sure that you have correctly followed the instructions for authors as given on the submission website.

*** PLEASE NOTE *** As part of the EMBO Press transparent editorial process initiative (see our Editorial at https://dx.doi.org/10.1038/msb.2010.72), Molecular Systems Biology publishes online a Review Process File with each accepted manuscripts. This file will be published in conjunction with your paper and will include the anonymous referee reports, your point-by-point response and all pertinent correspondence relating to the manuscript. If you do NOT want this File to be published, please inform the editorial office at msb@embo.org within 14 days upon receipt of the present letter.

Reviewer #1:

Wuyts et al used a combination of different omics techniques to characterize the response of a synthetic microbial community to 3 different drugs. These drugs were previously reported to have growth inhibitory effects in several members of the gut microbiome.
While I find the topic interesting and of potential interest for the community of systems biologists , I have several concerns. It is not entirely clear what is the main question the authors are trying to address here. Somehow the study falls short in going beyond collection of several omics data and observations.
- The main and most conclusive result is the possibility to use omics (i.e. metagenomics or transcriptomics) alternative to 16sRNA gene profiling to characterize the composition of the community.
It is difficult for me to understand the novelty of this result, or when, in order to quantify or characterize the composition of a microbial community one would opt for a different approach than 16sRNA. They authors should clarify this aspect in the discussion.

- Regarding the insights gain from the analysis of molecular profiles in drug-perturbed microbial communities it remains hard to judge what is that we learn. Overall, this entire part is very descriptive and speculative. Here some suggestions:
The discrepancy observed between previous inhibitory activities obtained from monoculture experiments and those presented here is in my view not adequately discussed. The authors invoke some general protective role from the community. They excluded drug degradation, but don't provide or test other alternatives. This difference in response to drugs is a central point to justify and interpret collected data. Is the state/physiology of bacteria cultivated alone so different from when they are cultivated all together? If so, can some of these differences explain the lower susceptibility? For example, could simply lower growth rates of the diverse members of the community when grown together explain the reduced inhibitory activity of the tested drugs?
It is also difficult to understand what are the results that are conclusive from those which might be mere artifacts. The metabolic changes observed in response to metformin are (if I understand correctly) caused by some MS-related artifacts. So why presenting them first? Or what not using other techniques which would be robust to such artifacts? What about the other drugs? How different are the observed changes from previous studies performing similar type of molecular profiling?
It is also not clear how the authors are correcting/normalizing these data for the observed changes in biomass. Can it be that the number of affected metabolites or proteins is largely dictated by the growth inhibitory activity? Specifically, I'm referring to chlorpromazine.
Overall, I find that, while it seems an obvious next step to investigate the response of microbial communities to drug perturbations, in its present form, this study presents little new biological insights and it remains difficult to judge whether the presented appraoch can pave the way to new strategies to understand drug response at the community level.

Reviewer #2:

This is a review of a manuscript entitled "Consistency across multi-omics layers in a drug-perturbed gut microbial community" authored by Wuyts, et al. In this manuscript, Wuyts et al. integrated multi-omics data sets to investigate the responses of a synthetic community to three non-antibiotic drugs (chlorpromazine, metformin, and niclosamide). Many studies of gut microbial communities integrate multiple levels of omics data, yet few have systematically evaluated the concordance and unique insights provided by these individual sets of data. By focusing on a defined synthetic community in vitro, authors of the current study provide clear insights into the taxonomic and functional information provided by 16S rRNA profiling, shotgun metagenomics, metatranscriptomics, metaproteomics, and metabolomics. The overall conclusions are that techniques that provide species-level abundance estimates generally have high concordance and that functional tools (metagenomics, metatranscriptomics, metaproteomics, and metabolomics) provide complementary insights into microbial community functions. In general, this manuscript is well-written and has a clear logical flow to guide the reader through their experimental design and data analysis. The presentation of the results are clear and easily understandable. While overall enthusiastic about this study, I have a few suggestions that if addressed, would result in an improved manuscript. Please find major and minor comments below:

Major comments:
1. Metabolomics data processing. In the manuscript, the authors followed Analytical Chemistry paper (Anal. Chem. 2011, 83, 18, 7074-7080), which only used m/z for metabolite identification. This approach was originally designed for a flow-injection rather than the current LC-MS approach. It would have a potentially high false positive rate in metabolite annotation and high redundancy. This likely explains why many redundancies exist in the metabolomics data (Supplementary Figure 8). Ideally, the

authors would use additional lines of support (e.g., MS/MS and/or retention time) to improve the confidence of metabolite identification. The authors should acknowledge the limitations to their approach and clearly state that metabolite annotation confidence is modest (e.g., level 3 according to the metabolomics society).
2. Following comment above, it is not clear how the authors handle metabolite redundancy (one feature with multiple metabolite annotations, e.g. supplementary figure 8). If all of them are directly counted in the pathway enrichment analysis, it would likely lead to overrepresentation of pathways. This should be addressed.
3. Figures 3B and 4B. Why did the authors elect to include "all conditions and timepoints" in these analyses? How would the data look if they were split out across timepoints?

Minor comments:
1. The authors performed two independent experiments (Run A and Run B). The biological replication and evaluation of the reproducibility of the techniques are overall concordant, however, several inconsistencies in the result do exist. As the authors indicate (page 9, lines 275-276), obvious abundance differences exist between run A and run B. It may decrease the reproducibility confidence of multi-omics results. How do these differences affect the results in this study? Does this suggest that more biological replicates would be required in future studies?
2. Page 12, lines 359-360: Suggest changing "an increased metabolization of nitrogen" to "increased nitrogen metabolism"
3. Page 14, line 389. As the author mentioned in the introduction, different omics change at different time scales. It is interesting to observe the delayed fold-change correlation between metatranscriptomics and metaproteomics. Did the authors observe the corresponding concordant changes/correlations at the functional pathway level? If not, would the authors discuss the possible reasons for this?
4. I would suggest the authors provide some general numbers (e.g. number of genes, proteins, metabolites) of each omics level. This would help readers get a general sense of the results.
5. It would be clearer if the authors could label the two clusters in Figure 2E.
6. The authors refer to "polysaccharides" generally in their interpretation of the data. However, the only polysaccharide included in the medium is soluble starch which is relatively well defined. I suppose it is also possible that yeast mannans could be present from the yeast extract. Do the proteomics and metatranscriptomics data provide insights in the specific polysaccharide utilization loci that are highly expressed by Bacteroides spp. present in the community?
7. Following up on the comment above, I believe some extra thought should be given to the interpretation of "increased polysaccharides" from the metabolomics data. First, Figure 4 shows putative annotations for hexose containing oligo-saccharides, not polysaccharides. Second, Bacteroides are not the only species within the community that are known to breakdown polysaccharides such as starch. If the oligosaccharides are increased, might that suggest that polysaccharides are indeed being depolymerized, but that the resulting oligosaccharides are not being efficiently consumed?

Reviewer #3:

The manuscript by Wuyts et al studied the effects of three drugs (chlorpromazine, metformin, niclosamide) on a synthetic microbial community composed of 32 human gut bacteria. The study collected microbial samples at multiple time points after drug exposure and profiled the microbial communities using five different omics techniques, including 16s amplicon sequencing, metagenomics, metatranscriptomics, proteomics, and metabolomics. The focus of the study was to investigate the consistency between the five omics results. The study found that the five omics techniques produced consistent results on community compositions, whereas the functional profiles from different omics were more diverse. Among the three drugs tested, chlorpromazine showed the largest perturbation on the microbial communities, while metformin and niclosamide showed minor perturbations. The study further observed stress response induced by chlorpromazine and proposed molecular processes of the stress response based on the multi-omics data.
The investigation into multi-omics consistency is novel. The study is thoroughly conducted, and the manuscript is well-written and clearly communicated. However, the reviewer has some major concerns over the compound prioritization and identification in the metabolomics part. Note that the reviewer is a metabolomics scientist, so most of the comments focus on the metabolomics part of the study.

Specific comments:
1. Line 692-708: The reviewer does not agree with the metabolite annotation strategy used here. Annotations based on MS1 only produce too many false-positives. The reviewer strongly suggests the authors collect MS2 data and identify metabolites based on MS1 and MS2. The metabolite identification could also be improved by using databases more specific to microbial metabolites (e.g., MiMeDB) instead of HMDB. At minimum, the limitation in metabolite annotation should be clearly stated in the manuscript.
2. Line 706-708: To the reviewer, neither of the criteria listed (high correlation with TIC or large intensity) justifies an ion to be annotated more correctly. MS2 spectra need to be collected to improve identification confidence.
3. Line 127-128 and 554-556: I suggest listing some intestinal concentrations previously reported for the three drugs. It helps justify the relevance of the concentrations used in this study.
4. Line 237-240, 335-337, 556-572: Please include description of the non-bacterial control samples in the Method section. They are an important set of controls. It is interesting to see in Figure S8 that metformin addition changed the detected metabolite abundance in the culture media while the other two drugs did not. The author suggested that metformin can affect the mass

spectrometry detection of these metabolites. Is it also possible that metformin could chemically react with these metabolites? Also, metabolites with changed abundance in non-bacterial controls should be excluded from downstream pathway analysis.

5. Line 240-241, 576-577: Why only the excreted metabolites were analyzed? Is there any justification to not extract the cell pellets for metabolomics analysis?

6. Line 682: Is there any reason to use an isocratic gradient for metabolomics analysis? Does the isocratic method provide good enough coverage for metabolites of different polarities?

We thank all three reviewers for their constructive comments on our manuscript, which we answer in detail below.

Reviewer #1:

Wuyts et al used a combination of different omics techniques to characterize the response of a synthetic microbial community to 3 different drugs. These drugs were previously reported to have growth inhibitory effects in several members of the gut microbiome.

While I find the topic interesting and of potential interest for the community of systems biologists , I have several concerns. It is not entirely clear what is the main question the authors are trying to address here. Somehow the study falls short in going beyond collection of several omics data and observations.
- The main and most conclusive result is the possibility to use omics (i.e. metagenomics or transcriptomics) alternative to 16sRNA gene profiling to characterize the composition of the community.
It is difficult for me to understand the novelty of this result, or when, in order to quantify or characterize the composition of a microbial community one would opt for a different approach than 16sRNA. They authors should clarify this aspect in the discussion.

In this study, we had three main research questions: 1) How do the different omics methods perform in capturing changes in microbial communities in response to perturbations? 2) In which time frame do drugs cause perturbations on the bacteria, as visible in genomes, transcripts, proteins, and metabolites? 3) Can we identify the drug's mechanism of action on the bacteria, or the bacteria's defensive responses? We tried to make this clearer to the reader by adding these specific research questions to the last paragraph of the introduction (page 5).

We also want to point out that our study results go beyond collecting omic data, as they provide novel insights into the effect of non-antibiotic drugs on microbial communities. While our results are inconclusive for metformin, we did find that niclosamide is being degraded by our microbial community. In the literature, this has been attributed to nitroreductases, but interestingly we do not find an upregulation of these enzymes, raising the possibility that nitroreductases that are already being expressed can detoxify the drug. In addition, we found that chlorpromazine has a huge effect on the community by depleting Bacteroidota species. We believe that chlorpromazine is acting as an inhibitor of their multi-efflux pump BmeB, which in combination with protein aggregation stress is causing the depletion of these species.

16S rRNA sequencing will generally lead to a genus-level resolution of the microbial community. Other methodologies such as metagenomics and metatranscriptomics allow for a species-level resolution or even strain level resolution. These methodologies therefore offer a better solution when studying communities with phylogenetically closely related members. Moreover, metatranscriptomics provides additional information on species transcriptional activity, which is essential to characterise functional changes in the community. We have added a paragraph in the discussion section to clarify this (page 15).

- Regarding the insights gain from the analysis of molecular profiles in drug-perturbed microbial communities it remains hard to judge what is that we learn. Overall, this entire part is very descriptive and speculative. Here some suggestions:

The discrepancy observed between previous inhibitory activities obtained from monoculture experiments and those presented here is in my view not adequately discussed. The authors invoke some general protective role from the community. They excluded drug degradation,

but don't provide or test other alternatives. This difference in response to drugs is a central point to justify and interpret collected data. Is the state/physiology of bacteria cultivated alone so different from when they are cultivated all together? If so, can some of these differences explain the lower susceptibility? For example, could simply lower growth rates of the diverse members of the community when grown together explain the reduced inhibitory activity of the tested drugs?

We thank the reviewer for these suggestions. Indeed, we were, unfortunately, not able to explain the absence of an effect for metformin with our current in-depth dataset. That is why we have chosen to report the observation and launch the hypothesis of a difference in physiology between culturing single species vs culturing a community of microbial species. However, since our current experiment was designed to compare different drug treatments to a baseline, which in this case was a community without any drug treatments, our experimental set-up did not include comparison to single species cultivation. Adding these experiments would go beyond the scope of this paper. However, we do agree that we did not adequately discuss this and therefore as the reviewer suggested, we now compare our results to the previous experiments with single species in more detail (page 12). As mentioned there, it has been shown that the physiology of bacteria cultivated alone can differ significantly than when cultivated in a community. We hope that this will lead to further investigation in the future, that can dive into the exact mechanisms of action at work and explain this discrepancy with a well designed experiment.

It is also difficult to understand what are the results that are conclusive from those which might be mere artifacts. The metabolic changes observed in response to metformin are (if I understand correctly) caused by some MS-related artifacts. So why presenting them first? Or what not using other techniques which would be robust to such artifacts? What about the other drugs? How different are the observed changes from previous studies performing similar type of molecular profiling?

We agree with the reviewer that this paragraph could have caused confusion. We have revised the paragraph (page 11), and refrained from presenting the artefacts first.

It is also not clear how the authors are correcting/normalizing these data for the observed changes in biomass. Can it be that the number of affected metabolites or proteins is largely dictated by the growth inhibitory activity? Specifically, I'm referring to chlorpromazine.

All measurements presented here are relative measurements, and therefore the total biomass does not have an effect on the data. (We added a sentence on this in the methods section, page 24.) The only exception is when we measure metabolites that are present in the medium. Here, our hypothesis is indeed that lower biomass of Bacteroidota species leads to a lower consumption of oligosaccharides. A uniform decrease in the overall biomass of the community could not explain the initially increased levels of saccharides shown in Fig. 4F.

Overall, I find that, while it seems an obvious next step to investigate the response of microbial communities to drug perturbations, in its present form, this study presents little new biological insights and it remains difficult to judge whether the presented appraoch can pave the way to new strategies to understand drug response at the community level.

We thank the reviewer for the critical remarks and suggestions. We now edited the manuscript in order to better clarify the biological and technical findings of the study and conclusions. We believe that the true power of multi-omics lies in its ability to generate novel hypotheses that should be validated in follow-up studies and hope that the reviewer agrees with this vision.

Reviewer #2:

This is a review of a manuscript entitled "Consistency across multi-omics layers in a drug-perturbed gut microbial community" authored by Wuyts, et al. In this manuscript, Wuyts et al. integrated multi-omics data sets to investigate the responses of a synthetic community to three non-antibiotic drugs (chlorpromazine, metformin, and niclosamide). Many studies of gut microbial communities integrate multiple levels of omics data, yet few have systematically evaluated the concordance and unique insights provided by these individual sets of data. By focusing on a defined synthetic community in vitro, authors of the current study provide clear insights into the taxonomic and functional information provided by 16S rRNA profiling, shotgun metagenomics, metatranscriptomics, metaproteomics, and metabolomics. The overall conclusions are that techniques that provide species-level abundance estimates generally have high concordance and that functional tools (metagenomics, metatranscriptomics, metaproteomics, and metabolomics) provide complementary insights into microbial community functions. In general, this manuscript is well-written and has a clear logical flow to guide the reader through their experimental design and data analysis. The presentation of the results are clear and easily understandable. While overall enthusiastic about this study, I have a few suggestions that if addressed, would result in an improved manuscript. Please find major and minor comments below:

Major comments:
1. Metabolomics data processing. In the manuscript, the authors followed Analytical Chemistry paper (Anal. Chem. 2011, 83, 18, 7074-7080), which only used m/z for metabolite identification. This approach was originally designed for a flow-injection rather than the current LC-MS approach. It would have a potentially high false positive rate in metabolite annotation and high redundancy. This likely explains why many redundancies exist in the metabolomics data (Supplementary Figure 8). Ideally, the authors would use additional lines of support (e.g., MS/MS and/or retention time) to improve the confidence of metabolite identification. The authors should acknowledge the limitations to their approach and clearly state that metabolite annotation confidence is modest (e.g., level 3 according to the metabolomics society).

We thank the reviewer for their feedback on the employed metabolomics approach. Indeed our method is very similar to the one in the paper we cited, except for some differences in analytical instrumentation (e.g. we use an orbitrap instrument while Fuhrer et al. use a Q-TOF, and we use an LC pump / autosampler combination from Thermo rather than the Agilent/Gerstel setup from the Fuhrer et al. paper). Other than that, as in the cited paper, we also use flow injection and hence also have accurate m/z as the sole information available for tentative metabolite identification. We have re-phrased the respective sections in the manuscript (page 22f) to make this clearer to the journal's readership. We also added further statements on the limitations of this approach to the results and methods section (page 9, top; page 23).

2. Following comment above, it is not clear how the authors handle metabolite redundancy (one feature with multiple metabolite annotations, e.g. supplementary figure 8). If all of them are directly counted in the pathway enrichment analysis, it would likely lead to overrepresentation of pathways. This should be addressed.

We thank the reviewer for pointing out the issue with metabolite redundancy. Indeed, we perform pathway enrichment analysis on ion features, and not metabolite features: if one ion is annotated as multiple metabolites from the same pathway, it is counted only once for the enrichment analysis. To clarify this, we added the following text to the methods section: "For metabolomics, pathway enrichment analysis was performed for ion features rather than

metabolite features (e.g. if one ion is annotated to two or more metabolites from the same pathway, it is counted only once in pathway enrichment analysis).” (page 26)

3. Figures 3B and 4B. Why did the authors elect to include "all conditions and timepoints" in these analyses? How would the data look if they were split out across timepoints?

Unfortunately our sample sizes did not allow us to split out the analysis for each time point separately. We only had two runs of the experiment, leading to two independent samples per time point, which did not give us enough statistical power. We therefore opted to group them together. For the analysis underlying Figure 3B we used time as a covariate for which we adjusted the ANCOM analysis while testing the differences between species abundances in drug perturbed samples versus control. This analysis thus summarises the changes in community composition that happen over time. We added a clarification to the corresponding methods section. For reader’s reference, we provide Figure 3A where the dynamics of the changes in community composition is depicted over time.

For the analysis underlying Figure 4B, we chose to perform pathway enrichment analysis for features using only one fold change value per feature (with the maximum absolute change over time). We chose to perform analysis in this way for two reasons: 1) there are few changes observed in the early time points, as depicted in Figure 4A, therefore pathway enrichment analysis is not yielding results; 2) most of the observed changes have ascending or descending dynamics over time, and thus can be summarised with one number, which we chose to be maximum absolute change. We clarified these analysis choices in the methods section (page 26).

Minor comments:

1. The authors performed two independent experiments (Run A and Run B). The biological replication and evaluation of the reproducibility of the techniques are overall concordant, however, several inconsistencies in the result do exist. As the authors indicate (page 9, lines 275-276), obvious abundance differences exist between run A and run B. It may decrease the reproducibility confidence of multi-omics results. How do these differences affect the results in this study? Does this suggest that more biological replicates would be required in future studies?

While there were some differences between the two runs, the overall shifts followed similar trends and significant results did not contradict each other between runs (Fig. 3B). That said, due to the complex nature of multi-omics experimental setups, multiple replicates, both biological and technical, should always be considered. Here, we invested resources in the breadth of the multi-omics measurements. In a more focused paper, using e.g. only metatranscriptomics and metabolomics, three or more biological replicates would certainly be advisable.

2. Page 12, lines 359-360: Suggest changing "an increased metabolization of nitrogen" to "increased nitrogen metabolism"

Agreed! We changed the wording as suggested (page 12).

3. Page 14, line 389. As the author mentioned in the introduction, different omics change at different time scales. It is interesting to observe the delayed fold-change correlation between metatranscriptomics and metaproteomics. Did the authors observe the corresponding concordant changes/correlations at the functional pathway level? If not, would the authors discuss the possible reasons for this?

We thank the reviewer for this suggestion. However, since we only collected extracellular metabolomics data, we cannot perform this comparison of functional pathway changes across transcriptomics, proteomics and metabolomics.

4. I would suggest the authors provide some general numbers (e.g. number of genes, proteins, metabolites) of each omics level. This would help readers get a general sense of the results.

Most of the values alluded to are mentioned in page 8. Specifically, the total number of protein-coding genes, transcripts and predicted proteins are included. These correspond to the subset that could be identified across different omics levels. We have additionally added the number of detected metabolites in the manuscript (page 9, top).

5. It would be clearer if the authors could label the two clusters in Figure 2E.

While considering this suggestion, we have realised that we should replace the term “cluster” by “group”, as transcript abundance from metatranscriptomics correlates with all other omics measurements. It would therefore be too restrictive to only consider it part of one cluster, but it should rather be considered to belong to both groups. We have updated the text accordingly (page 9, bottom), and have refrained from adding cluster labels to Fig. 2E.

6. The authors refer to "polysaccharides" generally in their interpretation of the data. However, the only polysaccharide included in the medium is soluble starch which is relatively well defined. I suppose it is also possible that yeast mannans could be present from the yeast extract. Do the proteomics and metatranscriptomics data provide insights in the specific polysaccharide utilization loci that are highly expressed by Bacteroides spp. present in the community?

We thank the reviewer for this interesting question. First, we do want to clarify that as a complex medium mGAM includes extracts of meat, liver and yeast where glycogen can be found, but also other animal polysaccharides in smaller amounts, next to starch. Secondly, while this question is definitely interesting, we believe that a thorough analysis of all polysaccharide utilization loci (PUL) was out of the scope of the paper. For the readers interested in specific genes or groups of genes, we provide the files necessary to perform such analysis on FigShare. For example the following files contain information on gene expression across conditions:

- metatranscriptomics_gene_counts_normalised_per_species.tsv : The metatranscriptomic gene count, normalised per species
- 32_genomes.prokka.eggnog_annotations.gff: The Prokka annotations of each gene, which can be used to screen for PUL related genes

7. Following up on the comment above, I believe some extra thought should be given to the interpretation of "increased polysaccharides" from the metabolomics data. First, Figure 4 shows putative annotations for hexose containing oligo-saccharides, not polysaccharides. Second, Bacteroides are not the only species within the community that are known to breakdown polysaccharides such as starch. If the oligosaccharides are increased, might that suggest that polysaccharides are indeed being depolymerized, but that the resulting oligosaccharides are not being efficiently consumed?

We thank the reviewer for pointing out that there might be an interplay between species depolymerizing polysaccharides and consuming the resulting oligosaccharides. Our data does not allow us to test this hypothesis directly, as we do not have the measurements of polysaccharides, and the time resolution of the sampling might be not frequent enough to capture polysaccharide utilisation dynamics. We apologise that our use of the term

“polysaccharides” was misleading; we changed it to “oligosaccharides” wherever we refer to di- tri- and tetrasaccharides in the text (page 14).

Reviewer #3:

The manuscript by Wuyts et al studied the effects of three drugs (chlorpromazine, metformin, niclosamide) on a synthetic microbial community composed of 32 human gut bacteria. The study collected microbial samples at multiple time points after drug exposure and profiled the microbial communities using five different omics techniques, including 16s amplicon sequencing, metagenomics, metatranscriptomics, proteomics, and metabolomics. The focus of the study was to investigate the consistency between the five omics results. The study found that the five omics techniques produced consistent results on community compositions, whereas the functional profiles from different omics were more diverse. Among the three drugs tested, chlorpromazine showed the largest perturbation on the microbial communities, while metformin and niclosamide showed minor perturbations. The study further observed stress response induced by chlorpromazine and proposed molecular processes of the stress response based on the multi-omics data.
The investigation into multi-omics consistency is novel. The study is thoroughly conducted, and the manuscript is well-written and clearly communicated. However, the reviewer has some major concerns over the compound prioritization and identification in the metabolomics part. Note that the reviewer is a metabolomics scientist, so most of the comments focus on the metabolomics part of the study.

Specific comments:
1. Line 692-708: The reviewer does not agree with the metabolite annotation strategy used here. Annotations based on MS1 only produce too many false-positives. The reviewer strongly suggests the authors collect MS2 data and identify metabolites based on MS1 and MS2. The metabolite identification could also be improved by using databases more specific to microbial metabolites (e.g., MiMeDB) instead of HMDB. At minimum, the limitation in metabolite annotation should be clearly stated in the manuscript.

We thank the reviewer for their feedback on the employed untargeted metabolomics approach and associated metabolite annotation strategy. As the reviewer correctly points out, the approach we chose to apply is unable to assign metabolite names with certainty as we only have accurate m/z information available for annotation. The advantage of our very open approach, however, is that it enables generation of hypotheses from data in a fairly unbiased way that more complex methodologies would find challenging to replicate. For studies in which individual metabolites turn out to be critical to a study’s conclusion, we fully agree with the reviewer that further validation of their annotation through additional data (chromatographic retention time, MSMS, ion mobility, NMR spectroscopy etc) is essential. In our study, however, the identities of individual metabolites are less critical as we aggregate metabolites at a pathway level before drawing conclusions (e.g. Fig 2D) which takes ambiguous annotations into account. Similarly, comparison of metabolomics with other omics data (Fig 2E) is performed based on ions, not annotations, and hence conclusions are not influenced by metabolite annotation confidence. We therefore believe that systematic acquisition of MSMS spectra (whose annotation would depend on limited availability of authentic standards, or validated computational predictions) would not affect the conclusions of our manuscript.

Nonetheless, to ensure readers are aware of limitations of the employed metabolomics approach, we added a clarifying statement to the Results section of the manuscript (page 9, top). We also further expanded on these limitations in the associated Methods section (page 23f). Finally, whenever specifically referring to individual metabolite annotations in the

manuscript), we modified our wording to reflect modest annotation confidence (e.g.: “the higher abundance of ions tentatively annotated as oligosaccharides after chlorpromazine treatment”, page 14).

We thank the reviewer for pointing out MiMeDB as the new resource for microbial metabolite annotation. We annotated our dataset with MiMeDB as well: with MiMeDB, we could annotate 345 ions in negative mode (of which 128 new annotations) and 1805 ions in positive mode (of which 817 new annotations). We added the MiMeDB annotation table to our data repository on Figshare. MiMeDB is a very new database (first publication in this year’s NAR Database Issue), and is evidently still growing and being worked on. For example, KEGG identifiers are not available in the current set of download files. In the future, it will certainly become a valuable resource for this kind of analysis.

2. Line 706-708: To the reviewer, neither of the criteria listed (high correlation with TIC or large intensity) justifies an ion to be annotated more correctly. MS2 spectra need to be collected to improve identification confidence.

We agree with the reviewer that high correlation with TIC or large intensity do not justify an ion to be annotated more correctly. We provide the original unfiltered annotation tables for the detected ions in the data repository for readers interested in our dataset, which we now also note in the Methods section (page 24).

3. Line 127-128 and 554-556: I suggest listing some intestinal concentrations previously reported for the three drugs. It helps justify the relevance of the concentrations used in this study.

Thank you for the suggestion! The concentrations are known for metformin (1.5 mM) and chlorpromazine (25 µM), and we’ve added these (page 6).

4. Line 237-240, 335-337, 556-572: Please include description of the non-bacterial control samples in the Method section. They are an important set of controls. It is interesting to see in Figure S8 that metformin addition changed the detected metabolite abundance in the culture media while the other two drugs did not. The author suggested that metformin can affect the mass spectrometry detection of these metabolites. Is it also possible that metformin could chemically react with these metabolites? Also, metabolites with changed abundance in non-bacterial controls should be excluded from downstream pathway analysis.

Thank you for pointing out this. We added the description of the non-bacterial control samples in the Methods section (page 24). We agree with the reviewer that the metabolites with changed abundance in non-bacterial controls could be removed from downstream analysis. However, in our analysis we decided to keep all metabolites detected and annotated in bacterial samples for downstream analysis, since the change in abundance detected in the bacterial sample may reflect the bacterial effect on the metabolite even if it is also changing in the control sample (the effect might be additive or even opposite). For transparency of data interpretation, we plot the abundance of all individually discussed metabolites together with non-bacterial control samples, and provide the full metabolomics dataset in the associated data repository.

5. Line 240-241, 576-577: Why only the excreted metabolites were analyzed? Is there any justification to not extract the cell pellets for metabolomics analysis?

We acknowledge that ideally both supernatant and cell pellet would be analyzed. We used the cell pellet for the other omics methods (sequencing and proteomics, plus a failed attempt at cell counting, and a backup sample). In future iterations, we would try to find a way to

include the cell pellet in metabolomics as well, for example to detect intracellular metabolites or bioaccumulation of a drug. Nonetheless, it has been shown before that medium and the secreted metabolites are important for the interactions between members of the community. We have added an explanation on page 9.

6. Line 682: Is there any reason to use an isocratic gradient for metabolomics analysis? Does the isocratic method provide good enough coverage for metabolites of different polarities?

As this is a flow injection approach, there is no need to have complex gradients as there is no separation by interaction with a stationary phase that needs to be optimized for different analyte classes. We only require the LC pump to deliver samples to the mass spec, not to support chromatographic separation on a column.

7th Jun 2023

Manuscript Number: MSB-2023-11525R
Title: Consistency across multi-omics layers in a drug-perturbed gut microbial community

Dear Peer,

Thank you for sending us your revised manuscript. We have now heard back from the three reviewers who were asked to evaluate your revised study. As you will see below, reviewers #2 and #3 are satisfied with the performed revisions and support publication. Reviewer #1 is still concerned about the conclusiveness of the main findings. Since reviewers #2 and #3 are satisfied with how the points related to this issue have been addressed, we think that this concern of reviewer #1 does not preclude the publication of the study. However, reviewer #1 also still has some concerns regarding the metabolomics measurements and the potential effects e.g. of the biomass on these measurements and the related conclusions. We would ask you to address these remaining concerns by providing additional clarifications and/or data, in a minor revision.

We would also ask you to address some remaining editorial issues listed below.

- Our data editors have noticed some unclear or missing information in the figure legends, please see the attached .doc file. Please make all requested text changes using the attached file and *keeping the "track changes" mode* so that we can easily access the edits made.

- The keywords need to be reduced to 5.

- Please include callouts to Fig. EV2A-C.

- The Appendix Figures and Tables should be labeled as Appendix Table S1 and Appendix Figure S1-S8. Please update the callouts in the text accordingly.

Please resubmit your revised manuscript online, with a covering letter listing amendments and responses to each point raised by the referees. Please resubmit the paper **within one month** and ideally as soon as possible. If we do not receive the revised manuscript within this time period, the file might be closed and any subsequent resubmission would be treated as a new manuscript. Please use the Manuscript Number (above) in all correspondence.

Click on the link below to submit your revised paper.

https://msb.msubmit.net/cgi-bin/main.plex

Thank you for submitting this paper to Molecular Systems Biology.

Kind regards,

Maria

Maria Polychronidou, PhD
Senior Editor
Molecular Systems Biology

If you do choose to resubmit, please click on the link below to submit the revision online before 7th Jul 2023.

https://msb.msubmit.net/cgi-bin/main.plex

IMPORTANT:
Please note that corresponding authors are required to supply an ORCID ID for their name upon submission of a revised manuscript (EMBO Press signed a joint statement to encourage ORCID adoption).
(https://www.embopress.org/page/journal/17444292/authorguide#editorialprocess)
Currently, our records indicate that the ORCID for your account is 0000-0002-2627-833X.

Please click the link below to modify this ORCID:
Link Not Available

The system will prompt you to fill in your funding and payment information. This will allow Wiley to send you a quote for the article processing charge (APC) in case of acceptance. This quote takes into account any reduction or fee waivers that you may be eligible for. Authors do not need to pay any fees before their manuscript is accepted and transferred to the publisher.

As a matter of course, please make sure that you have correctly followed the instructions for authors as given on the submission website.

*** PLEASE NOTE *** As part of the EMBO Press transparent editorial process initiative (see our Editorial at https://dx.doi.org/10.1038/msb.2010.72 , Molecular Systems Biology will publish online a Review Process File to accompany accepted manuscripts. When preparing your letter of response, please be aware that in the event of acceptance, your cover letter/point-by-point document will be included as part of this File, which will be available to the scientific community. More information about this initiative is available in our Instructions to Authors. If you have any questions about this initiative, please contact the editorial office (msb@embo.org).

Reviewer #1:

Overall, the authors did not adequately attempt to address my concerns.
The novelties listed by the authors are so far mainly speculations. If niclosamide degradation (which apparently was already reported) or chlorpromazine effects on Bacteroidota species are really the two key main novel findings of this paper, I would expect more than just speculations.

Certainly I don't see how simple experiments (i.e. comparison to single species cultivation) that are anyway very similar to the already performed experiments, can go beyond the scope of this paper. Given what the authors claimed on what are the major take home messages of this paper, the discussed matter is at the core of this work.

The authors also did not provide reasons to trust their metabolomics based analyses. Several speculations are constructed on the basis of metabolomics analyses, yet it remains unclear whether these data are overall affected by major artifacts.

The fact that all measurements are relative doesn't mean that difference in biomass doesn't have an effect. Are samples compared to other samples with the exact same biomass? What about composition for the respective microbial species? If not, changes in biomass during the course of the treatments or across antibiotics can have a drastic impact on the experimental results and their interpretation.

As far as it is described in the materials and methods the same volume (1ml) of cell culture was collected. Hence the extracted biomass in each sample can be different and proportional to OD. I cannot find anywhere how this is taken into account.

This will be important in understanding also whether it is the depletion of Bacteroidota species
leading to a lower consumption of oligosaccharides, or whether the lower levels of oligosaccharides are simply reflecting the lower abundance of Bacteroidota and (indeed the metabolic rate is identical).

Reviewer #2:

The authors have thoughtfully addressed my queries in full. This manuscript is ready for publication.

Reviewer #3:

The authors have either addressed my comments in the revised manuscript, or provided adequate discussion on the limitation of their analysis. I have no further comments and recommended publication.

Reviewer #1:

Overall, the authors did not adequately attempt to address my concerns. The novelties listed by the authors are so far mainly speculations. If niclosamide degradation (which apparently was already reported) or chlorpromazine effects on Bacteroidota species are really the two key main novel findings of this paper, I would expect more than just speculations.

Certainly I don't see how simple experiments (i.e. comparison to single species cultivation) that are anyway very similar to the already performed experiments, can go beyond the scope of this paper. Given what the authors claimed on what are the major take home messages of this paper, the discussed matter is at the core of this work.

We appreciate the reviewer's suggestion to perform single-species experiments, however, we are not sure how such experiments would benefit the interpretation of our data. For chlorpromazine treatment, we have already compared the observed changes in community composition to the previously published single-species data on drug–bacteria interactions. Indeed, also in single-species experiments, chlorpromazine had a large negative effect on the growth of Bacteroidota representatives, which is reflected in our community data. Our study expands this knowledge to the community context by showing that we do not see strong protective or sensitizing community effects, nor degradation of chlorpromazine. For niclosamide, however, we hypothesise that community has a protective effect, since community composition hardly changes, despite the fact that niclosamide affects the growth of many bacteria in single-species experiments. Since niclosamide is degraded by the community (as demonstrated with metabolomics measurements), we hypothesise that the drug degradation by some species is the main factor causing community protection. We do believe that unravelling the exact mechanisms of protective and sensitizing community effects is an important question for further studies, and would like to point out that a detailed study including niclosamide has just recently been published by our colleagues including several authors of this study: Garcia Santamarina, Kuhn et al. BioRxiv 2023 https://www.biorxiv.org/content/10.1101/2023.06.13.544832v1.

The authors also did not provide reasons to trust their metabolomics based analyses. Several speculations are constructed on the basis of metabolomics analyses, yet it remains unclear whether these data are overall affected by major artifacts.

The fact that all measurements are relative doesn't mean that difference in biomass doesn't have an effect. Are samples compared to other samples with the exact same biomass? What about composition for the respective microbial species? If not, changes in biomass during the course of the treatments or across antibiotics can have a drastic impact on the experimental results and their interpretation.

As far as it is described in the materials and methods the same volume (1ml) of cell culture was collected. Hence the extracted biomass in each sample can be different and proportional to OD. I cannot find anywhere how this is taken into account.

This will be important in understanding also whether it is the depletion of Bacteroidota species leading to a lower consumption of oligosaccharides, or whether the lower levels of oligosaccharides are simply reflecting the lower abundance of Bacteroidota and (indeed the metabolic rate is identical).

We appreciate the Reviewer's concerns regarding the interpretation of metabolomics analysis. While interpretation of untargeted metabolomics in our study indeed remains speculative, it comes with the nature of untargeted metabolomics datasets, and we do not believe that our dataset is affected by major artifacts.

First of all, we would like to note that we perform analysis of cell-free sample supernatants and not of intracellular metabolites. The same volume (1 ml) for all samples is collected, centrifuged, and 450 µl of supernatant is used for metabolomics analysis, therefore there is no biomass extraction that needs to be taken into account during the sampling step. We now clarified this in the Methods section “Experimental set-up and sample collection” and “Metabolomics measurements” (lines 616 and 717 in the Word document).

We agree with the reviewer that the metabolite abundance in the culture medium will be affected by bacterial biomass: the more bacteria are in the medium, the more metabolites they can consume or produce. While we cannot directly distinguish that metabolite changes in the medium are affected by changes in bacterial biomass or changes in bacterial metabolic activity, we account for changes in biomass in our computational analysis: i) We perform quantile normalization of the metabolomics data, which makes metabolite distributions across samples have identical statistical properties. In this way, metabolites are ranked within each sample, and the total metabolite intensity distributions between samples are equalized. ii) For metatranscriptomics and metaproteomics, gene expression and protein abundances are normalised per species. Thus, the observed lower expression of genes belonging to the starch and sucrose metabolism pathway is likely reflecting a lower metabolic rate. To clarify these points, we edited the main text (end of the results section, lines 446 to 461 in the Word document).

6th Jul 2023

Manuscript number: MSB-2023-11525RR
Title: Consistency across multi-omics layers in a drug-perturbed gut microbial community

Dear Peer,

Thank you again for sending us your revised manuscript. We are now satisfied with the modifications made and I am pleased to inform you that your paper has been accepted for publication.

*** PLEASE NOTE *** As part of the EMBO Publications transparent editorial process initiative (see our Editorial at https://dx.doi.org/10.1038/msb.2010.72), Molecular Systems Biology publishes online a Review Process File with each accepted manuscripts. This file will be published in conjunction with your paper and will include the anonymous referee reports, your point- by-point response and all pertinent correspondence relating to the manuscript. If you do NOT want this File to be published, please inform the editorial office at msb@embo.org within 14 days upon receipt of the present letter.

Should you be planning a Press Release on your article, please get in contact with msb@wiley.com as early as possible, in order to coordinate publication and release dates.

LICENSE AND PAYMENT:
All articles published in Molecular Systems Biology are fully open access: immediately and freely available to read, download and share.

Molecular Systems Biology charges an article processing charge (APC) to cover the publication costs. You, as the corresponding author for this manuscript, should have already received a quote with the article processing fee separately. Please let us know in case this quote has not been received.

Once your article is at Wiley for editorial production you will receive an email from Wiley's Author Services system, which will ask you to log in and will present you with the publication license form for completion. Within the same system the publication fee can be paid by credit card, an invoice or pro forma can be requested.

Payment of the publication charge and the signed Open Access Agreement form must be received before the article can be published online.

Molecular Systems Biology articles are published under the Creative Commons licence CC BY, which facilitates the sharing of scientific information by reducing legal barriers, while mandating attribution of the source in accordance to standard scholarly practice.

Proofs will be forwarded to you within the next 2-3 weeks.

Thank you very much for submitting your work to Molecular Systems Biology.

Kind regards,

Maria

Maria Polychronidou, PhD
Senior Editor
Molecular Systems Biology

EMBO Press Author Checklist

Corresponding Author Name: Peer Bork
Journal Submitted to: MSB
Manuscript Number: MSB-2023-11525

USEFUL LINKS FOR COMPLETING THIS FORM
The EMBO Journal - Author Guidelines
EMBO Reports - Author Guidelines
Molecular Systems Biology - Author Guidelines
EMBO Molecular Medicine - Author Guidelines

Reporting Checklist for Life Science Articles (updated January
This checklist is adapted from Materials Design Analysis Reporting (MDAR) Checklist for Authors. MDAR establishes a minimum set of requirements in transparent reporting in the life sciences (see Statement of Task: 10.31222/osf.io/9sm4x). Please follow the journal's guidelines in preparing your
Please note that a copy of this checklist will be published alongside your article.

Abridged guidelines for figures
1. Data
The data shown in figures should satisfy the following conditions:
- the data were obtained and processed according to the field's best practice and are presented to reflect the results of the experiments in an accurate and unbiased manner.
- ideally, figure panels should include only measurements that are directly comparable to each other and obtained with the same assay.
- plots include clearly labeled error bars for independent experiments and sample sizes. Unless justified, error bars should not be shown for technical
- if n<5, the individual data points from each experiment should be plotted. Any statistical test employed should be justified.
- Source Data should be included to report the data underlying figures according to the guidelines set out in the authorship guidelines on Data

2. Captions
Each figure caption should contain the following information, for each panel where they are relevant:
- a specification of the experimental system investigated (eg cell line, species name).
- the assay(s) and method(s) used to carry out the reported observations and measurements.
- an explicit mention of the biological and chemical entity(ies) that are being measured.
- an explicit mention of the biological and chemical entity(ies) that are altered/varied/perturbed in a controlled manner.
- the exact sample size (n) for each experimental group/condition, given as a number, not a range;
- a description of the sample collection allowing the reader to understand whether the samples represent technical or biological replicates (including how many animals, litters, cultures, etc.).
- a statement of how many times the experiment shown was independently replicated in the laboratory.
- definitions of statistical methods and measures:
  - common tests, such as t-test (please specify whether paired vs. unpaired), simple χ2 tests, Wilcoxon and Mann-Whitney tests, can be unambiguously identified by name only, but more complex techniques should be described in the methods section;
  - are tests one-sided or two-sided?
  - are there adjustments for multiple comparisons?
  - exact statistical test results, e.g., P values = x but not P values < x;
  - definition of 'center values' as median or average;
  - definition of error bars as s.d. or s.e.m.

Please complete ALL of the questions below.
Select "Not Applicable" only when the requested information is not relevant for your study.

Materials

Newly Created Materials	Information included in the manuscript?	In which section is the information available? (Reagents and Tools Table, Materials and Methods, Figures, Data Availability Section)
New materials and reagents need to be available; do any restrictions apply?	Not Applicable	

Antibodies	Information included in the manuscript?	In which section is the information available? (Reagents and Tools Table, Materials and Methods, Figures, Data Availability Section)
For **antibodies** provide the following information:<br>- Commercial antibodies: RRID (if possible) or supplier name, catalogue number and or/clone number<br>- Non-commercial: RRID or citation	Not Applicable	

DNA and RNA sequences	Information included in the manuscript?	In which section is the information available? (Reagents and Tools Table, Materials and Methods, Figures, Data Availability Section)
**Short novel DNA or RNA including primers, probes**: provide the sequences.	Not Applicable	

Cell materials	Information included in the manuscript?	In which section is the information available? (Reagents and Tools Table, Materials and Methods, Figures, Data Availability Section)
**Cell lines:** Provide species information, strain. Provide accession number in repository **OR** supplier name, catalog number, clone number, and/**OR** RRID.	Not Applicable	
**Primary cultures:** Provide species, strain, sex of origin, genetic modification status.	Not Applicable	
Report if the cell lines were recently **authenticated** (e.g., by STR profiling) and tested for mycoplasma contamination.	Not Applicable	

Experimental animals	Information included in the manuscript?	In which section is the information available? (Reagents and Tools Table, Materials and Methods, Figures, Data Availability Section)
**Laboratory animals or Model organisms:** Provide species, strain, sex, age, genetic modification status. Provide accession number in repository **OR** supplier name, catalog number, clone number, **OR** RRID.	Not Applicable	
**Animal observed in or captured from the field:** Provide species, sex, and age where possible.	Not Applicable	
Please detail **housing and husbandry conditions**.	Not Applicable	

Plants and microbes	Information included in the manuscript?	In which section is the information available? (Reagents and Tools Table, Materials and Methods, Figures, Data Availability Section)
**Plants:** provide species and strain, ecotype and cultivar where relevant, unique accession number if available, and source (including location for collected wild specimens).	Not Applicable	
**Microbes:** provide species and strain, unique accession number if available, and source.	Yes	Appendix Table 1

Human research participants	Information included in the manuscript?	In which section is the information available? (Reagents and Tools Table, Materials and Methods, Figures, Data Availability Section)
If collected and within the bounds of privacy constraints report on age, sex and gender or ethnicity for all study participants.	Not Applicable	

Core facilities	Information included in the manuscript?	In which section is the information available? (Reagents and Tools Table, Materials and Methods, Figures, Data Availability Section)
If your work benefited from core facilities, was their service mentioned in the acknowledgments section?	Yes	Acknowledgements

Design

Study protocol	Information included in the manuscript?	In which section is the information available? (Reagents and Tools Table, Materials and Methods, Figures, Data Availability Section)
If study protocol has been **pre-registered, provide DOI in the manuscript**. For clinical trials, provide the trial registration number **OR** cite DOI.	Not Applicable	
Report the **clinical trial registration number** (at ClinicalTrials.gov or equivalent), where applicable.	Not Applicable	

Laboratory protocol	Information included in the manuscript?	In which section is the information available? (Reagents and Tools Table, Materials and Methods, Figures, Data Availability Section)
Provide DOI OR other citation details if **external detailed step-by-step protocols** are available.	Not Applicable	

Experimental study design and statistics	Information included in the manuscript?	In which section is the information available? (Reagents and Tools Table, Materials and Methods, Figures, Data Availability Section)
Include a statement about **sample size** estimate even if no statistical methods were used.	Yes	Methods
Were any steps taken to minimize the effects of subjective bias when allocating animals/samples to treatment (e.g. **randomization procedure**)? If yes, have they been described?	Not Applicable	
Include a statement about **blinding** even if no blinding was done.	Not Applicable	
Describe **inclusion/exclusion criteria** if samples or animals were excluded from the analysis. Were the criteria pre-established?<br><br>If sample or data points were omitted from analysis, report if this was due to attrition or intentional exclusion and provide justification.	Not Applicable	
For every figure, are **statistical tests** justified as appropriate? Do the data meet the assumptions of the tests (e.g., normal distribution)? Describe any methods used to assess it. Is there an estimate of variation within each group of data? Is the variance similar between the groups that are being statistically compared?	Yes	Methods

Sample definition and in-laboratory replication	Information included in the manuscript?	In which section is the information available? (Reagents and Tools Table, Materials and Methods, Figures, Data Availability Section)
In the figure legends: state number of times the experiment was **replicated** in laboratory.	Yes	Figures
In the figure legends: define whether data describe **technical or biological replicates**.	Yes	Figures

Ethics

Ethics	Information included in the manuscript?	In which section is the information available? (Reagents and Tools Table, Materials and Methods, Figures, Data Availability Section)
Studies involving **human participants**: State details of **authority granting ethics approval** (IRB or equivalent committee(s), provide reference number for approval.	Not Applicable	
Studies involving **human participants**: Include a statement confirming that **informed consent** was obtained from all subjects and that the experiments conformed to the principles set out in the WMA Declaration of Helsinki and the Department of Health and Human Services Belmont Report.	Not Applicable	
Studies involving **human participants:** For publication of **patient photos**, include a statement confirming that consent to publish was obtained.	Not Applicable	
Studies involving experimental **animals**: State details of **authority granting ethics approval** (IRB or equivalent committee(s), provide reference number for approval. Include a statement of compliance with ethical regulations.	Not Applicable	
Studies involving **specimen and field samples:** State if relevant **permits** obtained, provide details of authority approving study; if none were required, explain why.	Not Applicable	

Dual Use Research of Concern (DURC)	Information included in the manuscript?	In which section is the information available? (Reagents and Tools Table, Materials and Methods, Figures, Data Availability Section)
Could your study fall under dual use research restrictions? Please check biosecurity documents and list of **select agents and toxins** (CDC): https://www.selectagents.gov/sat/list.htm	Not Applicable	
If you used a select agent, is the security level of the lab appropriate and reported in the manuscript?	Not Applicable	
If a study is subject to dual use research of concern regulations, is the name of the **authority granting approval and reference number** for the regulatory approval provided in the manuscript?	Not Applicable	

Reporting

The MDAR framework recommends adoption of discipline-specific guidelines, established and endorsed through community initiatives. Journals have their own policy about requiring specific guidelines and recommendations to complement MDAR.

Adherence to community standards	Information included in the manuscript?	In which section is the information available? (Reagents and Tools Table, Materials and Methods, Figures, Data Availability Section)
State if relevant guidelines or checklists (e.g., **ICMJE, MIBBI, ARRIVE, PRISMA**) have been followed or provided.	Not Applicable	
For **tumor marker prognostic studies,** we recommend that you follow the **REMARK** reporting guidelines (see link list at top right). See author guidelines, under 'Reporting Guidelines'. Please confirm you have followed these guidelines.	Not Applicable	
For **phase II and III randomized controlled trials**, please refer to the **CONSORT** flow diagram (see link list at top right) and submit the CONSORT checklist (see link list at top right) with your submission. See author guidelines, under 'Reporting Guidelines'. Please confirm you have submitted this list.	Not Applicable	

Data Availability

Data availability	Information included in the manuscript?	In which section is the information available? (Reagents and Tools Table, Materials and Methods, Figures, Data Availability Section)
Have **primary datasets** been deposited according to the journal's guidelines (see 'Data Deposition' section) and the respective accession numbers provided in the Data Availability Section?	Yes	Data Availability
Were **human clinical and genomic datasets** deposited in a public access-controlled repository in accordance to ethical obligations to the patients and to the applicable consent agreement?	Not Applicable	
Are **computational models** that are central and integral to a study available without restrictions in a machine-readable form? Were the relevant accession numbers or links provided?	Not Applicable	
If publicly available data were reused, provide the respective **data citations in the reference list**.	Not Applicable	
